# Reconfigurable Magnetic Origami Actuators with On‐Board Sensing for Guided Assembly

**DOI:** 10.1002/adma.202008751

**Published:** 2021-05-09

**Authors:** Minjeong Ha, Gilbert Santiago Cañón Bermúdez, Jessica A.‐C. Liu, Eduardo Sergio Oliveros Mata, Emily E. Evans, Joseph B. Tracy, Denys Makarov

**Affiliations:** ^1^ Helmholtz‐Zentrum Dresden‐Rossendorf e.V. Institute of Ion Beam Physics and Materials Research Bautzner Landstrasse 400 Dresden 01328 Germany; ^2^ Department of Materials Science and Engineering North Carolina State University Raleigh NC 27695 USA; ^3^ Department of Physics Elon University Elon NC 27244 USA

**Keywords:** actuation, magnetic materials, origami, reconfigurable materials, sensors

## Abstract

Origami utilizes orchestrated transformation of soft 2D structures into complex 3D architectures, mimicking shapes and functions found in nature. In contrast to origami in nature, synthetic origami lacks the ability to monitor the environment and correspondingly adjust its behavior. Here, magnetic origami actuators with capabilities to sense their orientation and displacement as well as detect their own magnetization state and readiness for supervised folding are designed, fabricated, and demonstrated. These origami actuators integrate photothermal heating and magnetic actuation by using composite thin films (≈60 µm thick) of shape‐memory polymers with embedded magnetic NdFeB microparticles. Mechanically compliant magnetic field sensors, known as magnetosensitive electronic skins, are laminated on the surface of the soft actuators. These ultrathin actuators accomplish sequential folding and recovery, with hinge locations programmed on the fly. Endowing mechanically active smart materials with cognition is an important step toward realizing intelligent, stimuli‐responsive structures.

## Graphical abstract

Magnetic origami actuators equipped with highly compliant magnetic field sensors can monitor their shape and detect their magnetization state, making feedback control and guided assembly possible. These actuators are based on composite films of ferromagnetic microparticles embedded in shape‐memory polymer films. Photothermal heating from light and applied magnetic fields enable on‐demand reconfigurability.

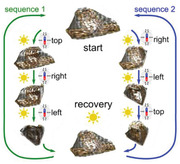

## 1 Introduction

Origami, the traditional art of paper folding, has drawn inspiration over centuries from patterns occurring in nature. A key aspect of origami is sequential folding, allowing plants and animals to fold and unfold wings, leaves, petals and self‐assembled organelles, ensuring their adaptability and survival in different environments.^[^ ^1^, ^2^, ^3^, ^4^ ^]^ Origami approaches are broadly applied for realizing smart actuators inspired by the behavior of living systems and their ability to respond to diverse physical and chemical stimuli, including electrical charges and dipoles, pressure, temperature, humidity, and magnetic fields.^[^ ^5^, ^6^, ^7^, ^8^, ^9^, ^10^, ^11^, ^12^, ^13^, ^14^, ^15^, ^16^, ^17^ ^]^ These mechanically active structures are typically designed to work within a predefined parameter range, outside of which they may fail to respond as desired. Endowing synthetic origami systems with the ability to detect environmental conditions and their own state mimics nature, enables feedback control, and enhances their ability to adapt to changes in the environment. Mechanically soft sensors are required that can adapt to motion and deformation during actuation are required for effective integration with origami. Standard approaches for soft actuators have focused either on rigid designs based on commercial electronics and pneumatic systems,^[^ ^18^ ^]^ or on small‐scale platforms with stimuli responsive materials.^[^ ^19^ ^]^ The former are too bulky to replicate the seamless and gentle folding patterns found in biological systems, and the latter lack sensors and thus feedback control for actively guiding their motion. Achieving soft, functional, and thin origami actuators requires a synergy between these two approaches, which can be mediated by using electronic skins (e‐skins), composite films or hydrogels. Recent works have achieved some steps toward this synergy by demonstrating intrinsically pliable strain,^[^ ^20^, ^21^ ^]^ curvature,^[^ ^22^, ^23^ ^]^ and optical^[^ ^24^ ^]^ sensors integrated onto soft actuators. Yet these examples focus on actuators made of single layers of material without multiple folds, and thus do not require motion tracking during assembly, as needed by origami. Detecting the position and orientation of various flaps or folds can be readily achieved by incorporating magnetosensitive e‐skins onto soft magnetic actuators, which detect the external or intrinsic (generated by the actuator) magnetic fields.

Specifically for magnetic soft actuators or magnetic soft robots^[^ ^1^, ^25^, ^26^, ^27^, ^28^, ^29^ ^]^ constructed from polymeric composites with embedded magnetic particles, changes in the magnetization state can dramatically affect actuation.^[^ ^24^, ^25^, ^30^, ^31^, ^32^, ^33^, ^34^, ^35^ ^]^ When such changes in the magnetic properties are purposeful and controlled, they can be highly beneficial for allowing the same structure to respond in new ways. The response of a magnetic actuator to an applied magnetic field is characteristic of the magnetization state of the composite, which is sensitive to both the process used for magnetizing the composite and to environmental exposures, particularly temperature. Heating the composite can affect the mechanics of the polymeric matrix and might lead to the redistribution of magnetic particles, thus modifying the state of the actuator. Changing the magnetization of the composite would alter and likely reduce magnetic torques and forces, resulting in a weaker bending response. Therefore, it is important to endow the actuators with the ability to detect their own magnetization state, so that guided assembly can be seamlessly achieved. This added capability improves the reliability and adaptability of magnetic actuators.

Here, we report the design and fabrication of ultrathin magnetic origami actuators with integrated magnetic field sensors. The lightweight and ultrathin (≈60 µm) origami actuators are based on a composite of magnetic NdFeB microparticles dispersed in a shape‐memory polymer matrix. The NdFeB microparticles serve a bifunctional role because they provide simultaneous responses to magnetic fields and light via photothermal heating. Due to the thinness of the origami actuators, they can be folded around 180° angles in a small magnetic field of about 100 Oe, followed by unfolding under illumination. Sensitivity to the orientation and displacement of parts of actuators is achieved by laminating a highly compliant, 3 µm‐thick magnetic‐field‐sensitive e‐skin on the soft origami actuators. The magnetic field sensors also allow perception of small stray magnetic fields emanating from the magnetic composites, permitting measurement of the magnetization state of the actuator (magnetized up or down vs demagnetized). Detection of the magnetization state makes possible real‐time adjustment of the field applied for actuation or detection of a failed actuator. Feedback control enables sequential processes and driving external devices, including a rotation stage and electromagnets, which we demonstrate for self‐guided assembly of a 4‐arm actuator. Furthermore, combining local illumination and magnetic fields for driving actuation facilitates writing the locations of hinges on the fly. By not requiring predefined hinges, no mechanical preprogramming of the sheet is necessary, which makes it fully reconfigurable for unfolding and then refolding into a different structure. We demonstrate reconfigurable folding without predefined hinges by assembling a planar sheet into a boat‐like structure following two different folding sequences. Sensors provide assessment of the current folding state and the ability to control the sequence of folding. This system illustrates the synergy between soft actuators and soft electronics, paving the way for remote operation of autonomous soft robots. Achieving full autonomy and unconstrained movement of the prospective smart actuators will further require integrating untethered sensors. Enabling cognition for mechanically active smart materials has major implications for realizing intelligent stimuli responsive structures in shape‐programmable soft materials, soft robotics, and functional implants.

## 2 Results and Discussion

### 2.1 Foldable and Hingeless Magnetic Origami Actuators

**Figure 1:**
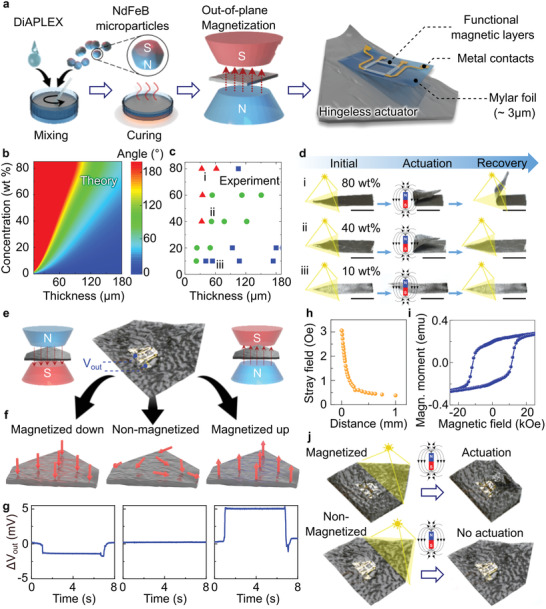
Magnetic origami actuators with magnetic field sensing capabilities. a) Schematic representation of the fabrication of magnetic origami actuators integrated with a compliant magnetic‐field sensor. b,c) Theoretical (b) and experimental (c) analyses of the folding angle of the DiAPLEX films for different film thicknesses and NdFeB microparticle loadings. In the experimental plot, the colors of the symbols represent bending approximate angles of 180° (red), 90° (green) and 0° (blue). The magnetic field used in the theoretical plot is 100 Oe (see experimental section). d) Photographs of actuation and recovery process of 60 µm‐thick actuator with different loadings of NdFeB microparticles: i) 80 wt%, ii) 40 wt%, and iii) 10 wt%. Composites with 80 wt% microparticle content (i) lost their shape‐memory properties. Composites with 10 wt% (iii) do not actuate. The composite with 40 wt% (ii) can achieve complete folding and retains the shape memory properties of DiAPLEX. Scale bar is 10 mm. e) Photograph of magnetic origami actuator integrated with a compliant magnetic field sensor. f) Illustration of the magnetic moment (thick red arrows) and the resultant stray field (dashed red arrows) of magnetized‐up/down and non‐magnetized magnetic origami actuators. g) The change of the readout voltage from the integrated compliant GMR sensors when assessing the magnetization state of magnetized down/up (left/right) and non‐magnetized (center) samples. h) Profile of the stray magnetic field of the magnetic origami, showing the dependence on the perpendicular separation from the surface of the actuator. i) Magnetization curve for a 60 µm‐thick actuator with 40 wt% NdFeB microparticles. j) Actuation behavior of magnetized (top) and non‐magnetized (bottom) actuators. After exposing the actuator to light and the magnetic field, only the magnetized foil folds according to its design.

Smart magnetic origami consists of two components, a bifunctional magnetic actuator combined with a mechanically imperceptible magnetic field sensor. The actuator is prepared by mixing magnetically hard NdFeB microparticles with a size of ≈5 µm with a thermoplastic polyurethane shape memory polymer (DiAPLEX), which is thermally activated, dissolved in tetrahydrofuran. The homogeneous mixture was poured into a mold defining the lateral layout of the sample (**Figure** **1**a). The thickness of the composite can be controlled in the range of 25–300 µm by pipetting different volumes of the mixture over a constant area and inspecting the resultant films by scanning electron microscopy (Figures S1 and S2, Supporting Information). The samples are magnetized in a magnetic field of 20 kOe oriented perpendicular to the plane of the actuator to provide deterministic actuation in external magnetic fields. Optical illumination drives photothermal heating, softening the composite and making it responsive to externally applied magnetic fields. This bifunctionality allows shape‐reconfigurable actuation without any predefined hinges. The location where folding takes place is determined on the fly by illuminating the area of interest with light and exposing the sample to magnetic fields from permanent magnets or an electromagnet.

To impart sensitivity to magnetic fields and feedback control of actuation, mechanically compliant high‐performance magnetic field sensors prepared on 3 µm‐thick Mylar foils are laminated on the magnetic actuator. The thinness of the Mylar foil ensures minimal perturbation of the mechanical properties, and the high compliance results in a stable response from the sensor upon bending. Two types of sensors were employed for different modes of operation. The strength and orientation of an in‐plane magnetic field are detected with angle sensors based on the giant magnetoresistance (GMR) effect. Out‐of‐plane magnetic fields are measured using anomalous Hall effect sensors and GMR sensors. A sensor would usually have one primary function, but in this work, sensors are used both for assessing the state of the magnetic actuator and measuring external fields to guide actuation.

Reconfigurable origami structures usually require bending up to 180° to define folds, which should also be fully recoverable. The bending angle of this soft actuator is determined by the external magnetic field, thickness of the composite film, and concentration of NdFeB microparticles in the DiAPLEX matrix (Figure 1b,c). For guiding experiments and more quickly achieving optimized experimental conditions, we have developed a phenomenological model for predicting the bending angle resulting from the balance between magnetic torques and elastic restoring torques. The model provides a closed analytical expression for the bending angle ϕ:

(1) $$\phi \;\;=\;\;6\frac{M_{\text{NdFeB}}\;f_{v}\left(1\;\;-\;\;f_{v}\right)}{E_{D}a^{2}}\;\;BL_{E}\;L_{M}$$

where an actuator of thickness *a* is assumed to possess a segment of constant curvature of length *L*_E_ and actuated segment of length *L*_M_ that is predominantly responsible for magnetic torques (Figure S3, Supporting Information) due to a magnetic flux density *B*; *M*_NdFeB_ and *f*_v_ are the respective magnetization and volume fraction of the NdFeB microparticles in the actuator, and *E*_D_ is Young's modulus of the DiAPLEX matrix.

The plot from theory in Figure 1b predicts ϕ across a range of parameters. For operation of our magnetic origami at about 47 °C, 180° folding may be expected for thicknesses below 80 µm and loadings of NdFeB microparticles above 50 wt%. The result depends on the temperature at which the actuation is performed. Increasing temperature reduces the Young's modulus of the DiAPLEX matrix, thus softening the actuator and enlarging the parameter range over which 180° folding is achieved (Figure S4, Supporting Information). For confirmation of these theoretical predictions, we fabricated and characterized rectangular films with lateral dimensions of 30 × 10 mm^2^, thickness of ≈25–300 µm, and NdFeB microparticle concentrations of 10–80 wt%. One of the short edges of each film was fixed on the center of a disc electromagnet, while the other end was free to move. Switching on the electromagnet generated a magnetic field (Figure S5, Supporting Information) that rotated the free end toward the center (Figure S6a, Supporting Information). To quantify folding under comparable heating conditions, the films were illuminated for 5 s, raising the temperature to about 45 °C (Figure S7, Supporting Information). The folding angle, defined between the stage and the plane formed by the free section of the film, was measured and compared for different film thicknesses and loadings of NdFeB microparticles (Figure S6b–d, Supporting Information). The experimental parameters that give ϕ of 180° qualitatively agree with theoretical predictions.

To ensure reversible and shape‐reconfigurable actuation, we investigated shape recovery of the magnetic composite films when increasing the NdFeB microparticle loading at a constant thickness of ≈60 µm. This thickness is sufficiently thin for 180° bending, while remaining substantially larger than the size of embedded NdFeB microparticles and yielding continuous films. We obtained the optimized film thickness and loading by comparing a series of films of different thicknesses and NdFeB loadings. At the highest loading of 80 wt%, films readily folded but failed to recover after illumination (Figure 1d‐i). Folding and recovery both occurred for films with 40 wt% (Figure 1d‐ii). Films with the loading further reduced to 10 wt% cannot be actuated with the same magnetic field (Figure 1d‐iii). At high loadings, NdFeB microparticles give a strong magnetic response and promote folding, but they also increase the elastic modulus of the composite. Moreover, less DiAPLEX is then present for driving shape recovery and imparting durability (Figure S6, Supporting Information). At high loadings, there is also significant agglomeration of the NdFeB microparticles (Figures S1 and S2, Supporting Information). Therefore, we selected a NdFeB microparticle concentration of 40 wt% for the following experiments, because it provides a good balance of actuation and recovery.

### 2.2 Self‐Detection of the Magnetization State

The highly compliant magnetic field sensors coupled to the magnetic origami actuator can detect their own stray fields. The control electronics use the measured signal to correlate the measured stray field with the actual magnetization state of the composite. Three magnetic origami actuators were prepared in different magnetization states to evaluate this capability. One film was not magnetized, and the others were magnetized up or down with a 20 kOe out‐of‐plane field (Figure 1e,f). A GMR sensor in a Wheatstone bridge configuration was integrated on the center of the actuator to detect the magnitude and direction of incoming stray magnetic fields. While GMR sensors are typically used as in‐plane sensors, when measured in an out‐of‐plane magnetic field and due to the hysteresis of the magnetic layer stack, the sensor reveals a linear signal change in the field range below the switching field (Figure S8a,b, Supporting Information). Utilizing this property, we can distinguish negative and positive out‐of‐plane magnetic fields at the site of the sensor by reading the output voltage (Figure 1e, center). For simplicity, we set the sensor readout of the stray fields from the non‐magnetized sample to zero. Increases and decreases of the measured voltage with respect to the non‐magnetized sample correspond to the up‐ and down‐magnetized actuators, respectively (Figure 1g and Movie S1, Supporting Information). We note that due to the non‐symmetric nature of the GMR sensor response when measured out‐of‐plane, the voltage output is non‐symmetric with respect to zero magnetic field (Figure 1g). The stray fields decrease with the distance from the surface of the actuator, with a maximum of 3 Oe at the surface (Figure 1h). The stray field is a measure of the spatial distribution of the remanent magnetic moment of the material (Figure 1i) and reflects the magnetization state of the actuator (Figure 1f). The actuation behavior strongly depends on the magnetization state of the composite. While magnetized samples fully actuate according to their design, the non‐magnetized actuator does not respond (Figure 1j and Movie S2, Supporting Information). It is important to note that the sensor readout (Figure 1g) unambiguously predicts the actuation behavior (Figure 1j). With this information about the current magnetization state, an operator could adjust the applied magnetic field for actuation or remagnetize the actuator in the desired state, thus allowing precise control over actuation even if magnetization changes arise. Depending on the extent of these changes, remagnetization may be required to yield the desired response even in strong magnetic fields or field gradients. Appropriately programmed control electronics could perform such adjustment autonomously, without human supervision. Autonomous capabilities can enhance many applications but could be particularly important for aerospace and deep‐water applications of smart actuators, which require autonomous operation.

### 2.3 Magnetic Field Sensors for Self‐Detection and Self‐Guided Assembly

**Figure 2:**
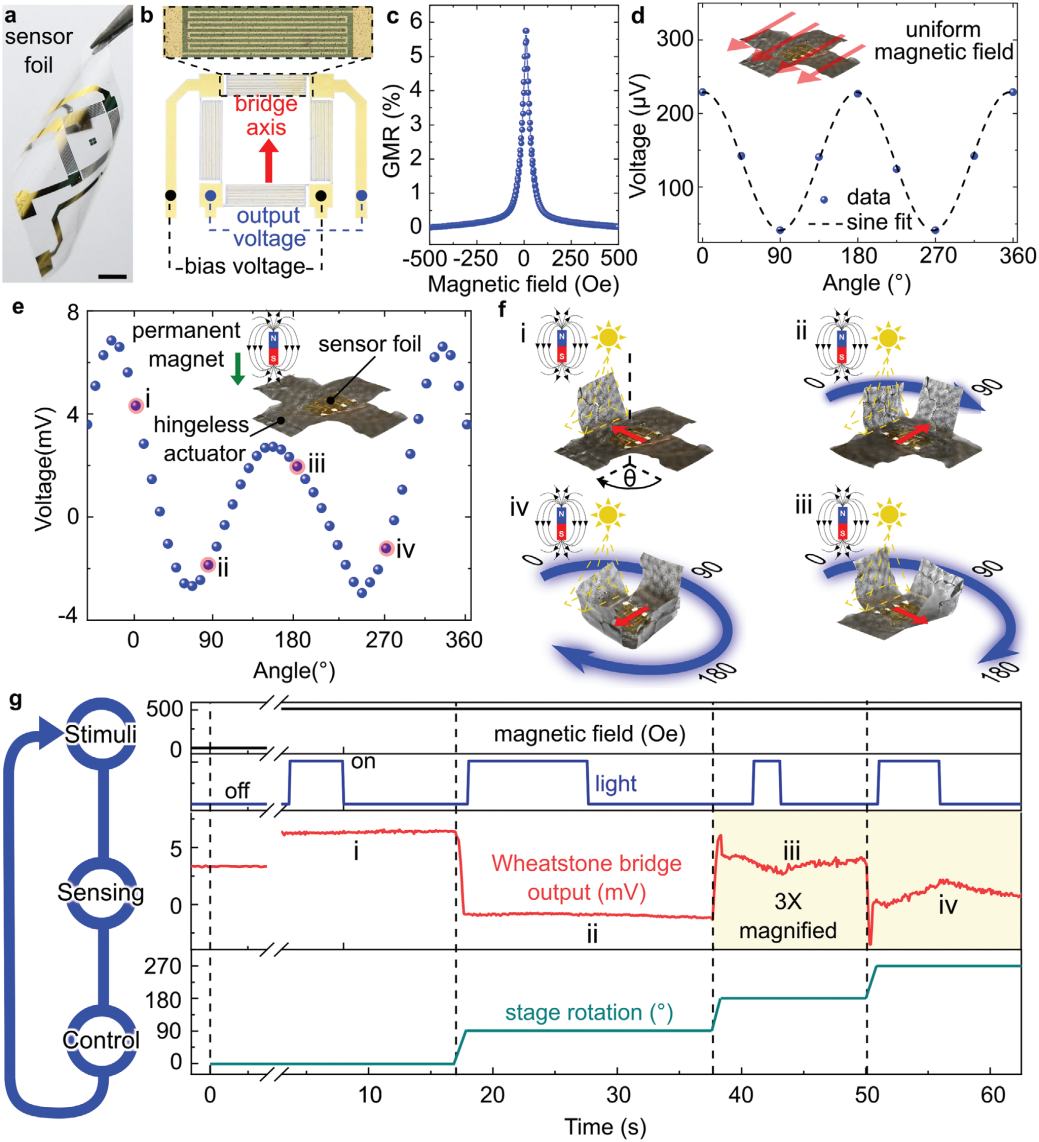
Self‐guided assembly of magnetic origami structures aided by compliant GMR sensors. a) Photograph of a highly compliant GMR sensor foil. Scale bar is 2 mm. b) Structure and connection scheme for angle sensor comprised of four GMR sensors arranged in a Wheatstone bridge configuration. Each sensor consists of a meander‐shaped Ni_81_Fe_19_/Cu multilayer stack (upper inset). Bias and output pins are staggered. c) GMR response of one of the sensor elements in the bridge as a function of the applied in‐plane magnetic field. d) Output voltage of the bridge as a function of the angle of a homogeneous in‐plane magnetic field with respect to the axis of the bridge. e) Output voltage of the bridge as a function of the angle of a spatially inhomogeneous magnetic field from a permanent magnet offset from the symmetry axis of the bridge. In panels (e) and (f), the location of the permanent magnet is indicated with a magnet bar sign. Important angles for our measurements are highlighted in red and denoted (i)–(iv). f) Assembly steps for folding the magnetic origami into a flower. i) A magnetic field source and a light source, are fixed on a particular location labeled 0°. Successive frames (i)–(iv) show the evolution of the fold as the stage rotates 90° during each step. g) Time evolution of the signals guiding the assembly process. The magnetic field and light are stimuli (upper 2 panels) detected by the sensor (third panel), which provides feedback for guiding rotation of the stage (lower panel).

The integrated magnetic field sensors enable feedback control for guiding the assembly of magnetic origami. To demonstrate this concept, a planar cross with four arms was designed that can undergo assembly into a flower (**Figure** **2**). Because of the bifunctionality of our actuators, the arms can bend or straighten when driven by light and an applied magnetic field. Compliant GMR sensors were prepared on a 3 µm‐thick Mylar foil (Figure 2a) and affixed to the origami with a poly(vinyl alcohol) (PVA) layer for guiding the assembly of the origami. Due to its inherent thinness, Mylar adheres very tightly and conforms to the curvy topography of the underlying DiAPLEX film, resisting delamination even when bent. The sensor can detect the orientation of in‐plane magnetic fields, which is ideal for monitoring assembly of the actuator. For this purpose, the sensor is located on the central tile of the actuator and is included in a feedback control loop programmed on a computer. The orientation sensor comprises four GMR sensors arranged in a Wheatstone bridge configuration with staggered contacts for bias and output (Figure 2b). Each sensor reveals a GMR ratio of 6% (Figure 2c). The low saturation field of ≈250 Oe for this GMR configuration allows reliable measurements even when using weak permanent magnets. The angular response of the sensor in a uniform in‐plane magnetic field has a periodicity of π (Figure 2d and Figure S8c, Supporting Information). By exposing the sensor to the inhomogeneous magnetic field of a permanent magnet offset from the rotation axis of the sensor, the angular response of the sensor changes, and the period becomes 2π (Figure 2e). Breaking the symmetry of the sensor response and the resulting longer period are beneficial for determining the relative orientation of the actuator over a large range of angles.

### 2.4 Self‐Guided Assembly of Magnetic Origami Actuators

Sequential control of actuation was achieved by intermittently switching on and off a light in the presence of a permanent magnet and using a computer program to process the output signals from the sensors. The position of the permanent magnet is kept fixed. The magnetic origami is rotated on a stepper motor. The angular orientation of the origami was determined with respect to the stationary magnetic field source by measuring the effect of the external magnetic field from the permanent magnet on the output voltage of the Wheatstone bridge. As the magnet approached a particular position, it disbalanced the bridge and allowed detection of the angular orientation of the magnetic origami actuator. Depending on the voltage received, the software commanded a stepper motor to rotate a certain number of steps to position the platform for the next assembly stage.

Once the desired orientation was achieved, an arm of the actuator was locally illuminated, softening the illuminated area and facilitating bending in an applied magnetic field. Such controlled bending requires precise knowledge of the orientation of the stage with respect to the light source. The permanent magnet and the light source were fixed over one of the arms, which was selected as 0° (Figure 2f‐i). The light was then switched on, causing the arm to bend upward toward the magnet. When the arm reached a vertical orientation, the light was switched off, and this configuration was maintained until the preprogrammed computer driving the rotation stage supporting the actuator commanded rotation in a 90° increment with feedback from the orientation sensor. Relative rotations of 90° (Figure 2f‐ii), 180° (Figure 2f‐iii) and 270° (Figure 2f‐iv) with respect to the 0° reference correspond to different voltage outputs of the Wheatstone bridge (Figure 2e). The response is characterized by two peaks: one of higher magnitude, which arises when the magnet approaches either of the dominant resistors of the bridge (those connected to ground) and leads to the maximum signal change. The other peak appears when the magnet approaches either of the remaining resistors, which have a lesser effect on the total output voltage and thus yield a smaller variation overall. A typical time trace for the actuation process is presented in Figure 2g, where rotating the stage triggers a change in the sensor output voltage. After each rotation event, the light is switched on for controlled folding of the arm. As seen in the upper panel of Figure 2g, some arms might require longer or shorter illumination times due to varying microparticle concentrations or arm geometries in the actuator. At the end of each folding step, the orientation sensor provides feedback‐controlled rotation of the stage before switching on illumination to lift the next arm. The entire assembly sequence is shown in Movie S3, Supporting Information.

### 2.5 Reconfigurability and Sequential Folding of Magnetic Origami Actuators

Foil‐like magnetic origami actuators can be folded without the need for predefined hinges, thus enabling numerous configurations starting from a flat state, in the spirit of pluripotent origami.^[^ ^36^, ^37^ ^]^ In principle, many equivalent configurations are possible, though the location of a hinge defined by illumination and the shape of the origami would also affect the bending behavior. Spatially controlled illumination and application of magnetic fields allow instantaneous customization of the folding process. In such conformationally flexible actuators, control and verification of the folding sequence are critically important for reproducible, sequential actuation.

**Figure 3:**
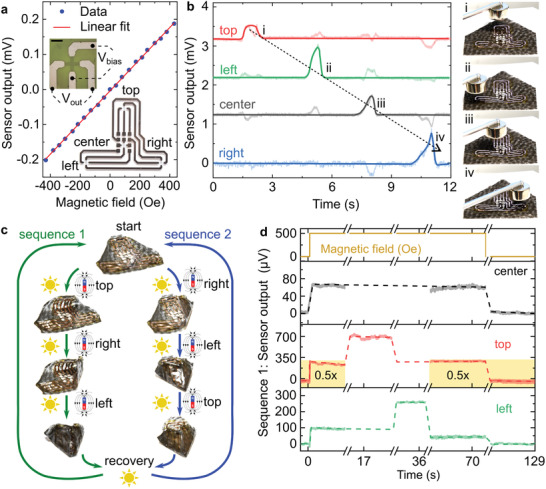
Sequential folding monitored by compliant anomalous Hall effect sensors. a) Output voltage of a single compliant anomalous Hall effect sensor as a function of the magnetic field applied perpendicular to the sensor plane. The output of the sensor is read transversally to the bias voltage (upper inset, scale bar is 1 mm). Four sensors are connected in series, distributed on the foil support at the top, center, right, and left positions (lower inset). b) Time trace of the outputs of the sensors in response to a hovering permanent magnet. As the magnet is moved above the top (i), left (ii), center (iii), and right (iv) sensors, the voltage changes in the sensors detect its presence. c) Illustration of two actuation sequences for assembly of the magnetic origami without predefined hinges. From the initial state (start), selective illumination guides the actuator to fold first its top flap or not. Folding it last (sequence 2) achieves a more defined final state in contrast with sequence 1. Illumination in the absence of magnetic fields recovers the initial, planar state of the origami. d) Output of the anomalous Hall effect sensors and the externally applied magnetic field (uppermost panel) as a function of time. The sensor in the center (second panel from the top) serves as a reference and does not change after turning on the magnet. The left and top sensors (last two panels) present a specific output voltage before actuation, reach a new steady‐state voltage after folding, and then recover their initial state.

A boat‐like actuator layout demonstrates the principles of hingeless sequential folding, recovery, and reconfiguration. The layout features left, right and top flaps. The top flap can bend over or below the other two flaps. A series of four compliant anomalous Hall effect sensors sensitive to out‐of‐plane magnetic fields were integrated with this hingeless magnetic origami actuator. Each sensor consists of a thin‐film metallic cross, biased with a constant current, and reads an output voltage transverse to the current (**Figure** **3**a, upper inset). The sensors were patterned on their carrier foil with one of the sensors placed as a reference in the center, while the remaining three were located at the corners for detecting the shape changes upon actuation, hence monitoring assembly of the origami structure (Figure 3a, lower inset). The anomalous Hall sensors displayed a linear dependence on the external magnetic field with a sensitivity of 500 µV kOe^–1^ (Figure 3a). These ultrathin anomalous Hall sensors do not significantly impede bending of the origami. Each of the sensors can respond to the magnetic field source located in its vicinity, as validated by sweeping a permanent magnet over the sensor array and recording the time trace of the sensor responses (Figure 3b). The appearance of the peak in the time trace allows deduction of where the magnet was placed and when it was moved above each of the flaps (Figure 3b‐i‐iv and Movie S4, Supporting Information). Monitoring the actuation of each flap is crucial for folding the boat structure in the proper sequence. The sequence of actuation determines the order in which the overlapping flaps are stacked. Thus, the order of folding can be ascertained from the signal of the anomalous Hall sensors.

We devised two sequences for demonstrating sequential folding guided by the anomalous Hall sensors (Figure 3c and Movies S5 and S6, Supporting Information). Assembly can be performed using an electromagnet (Movie S5, Supporting Information) or a permanent magnet (Movie S6, Supporting Information), giving qualitatively similar results. The magnetic field profile should be adjusted to pull the flaps toward the center. Folding is triggered by illuminating a selected flap. Sequence 1 started from the top flap, followed by the right and left flaps. In this sequence, the final shape is more compactly folded, as the larger top flap is kept in place by the right and left flaps (Figure 3c, left). In contrast, in sequence 2, the top flap is folded last and remains open, as it cannot be locked underneath the left and right flaps (Figure 3c, right). The sensors supervised sequential folding and measured whether a specific fold was completed or not, according to relative changes in their voltage output, presented in Figure 3d for sequence 1. The sensor on the center panel remained fixed during actuation and served as a reference for the other sensors (Figure 3d, second plot from the top). The angular orientation of the sensors on the top and left flaps changed during folding, modifying the out‐of‐plane component of the magnetic field they detected. Switching the electromagnet on increased the voltage in all the sensors simultaneously.

When the top flap was illuminated and lifted, its output voltage further increased as the sensor moved from a lower to a higher magnetic flux density region (Figure 3d, plot for top flap). The sensor mounted on the left flap experienced similar signal change upon illumination yet later in the sequence (Figure 3d, plot for left flap). Detection using the sensor mounted on the right flap is discussed in Figure S9 (Supporting Information). We note that despite the symmetrical placement of the sensors, not all of them experience the same magnetic field due to the complexity of the magnetic field profile generated by the underlying electromagnet. Furthermore, the folding of the flaps follows different trajectories for each of the sensors, which also results in different orientations or inclinations with respect to the magnetic field. Anomalous Hall effect sensors detect the magnetic field component perpendicular to the actuator surface during the folding process (Figure S10a, Supporting Information). Taking into account the complexity of the magnetic field of the electromagnet (Figure S5, Supporting Information), the magnitude of the magnetic field component normal to the sensor surface changes non‐monotonically upon actuation revealing a pronounced maximum (Figure S10b, Supporting Information). The presence of this maximum on the angular dependence of the normal component of the magnetic field at the sensor location agrees with the presence of the maximum appearing in the time evolution of the voltage signals measured by the anomalous Hall effect sensors in Figure 3d. Switching off the electromagnet and illuminating the whole structure drives recovery of the initial flat shape, and the signals from the sensors return to their initial state. Hence, measuring the output voltages from the sensors enables both monitoring and guiding sequential folding processes.

**3. Conclusion**

Magnetic origami actuators equipped with magnetic field sensors can guide their own assembly, by performing self‐supervised sequential folding and detection of their own magnetization state. These traits bring magnetic actuators closer to the full functionality of origami in nature, and self‐detection is an example of building structural health monitoring into actuators. Equipping photothermally and magnetically actuated shape memory polymer composites with highly compliant magnetosensitive electronic skins enables magnetic cognition and feedback control. Use of light for photothermal heating makes it possible to write hinges on the fly that are erased during shape recovery and can be subsequently reprogrammed, unlike predefined hinges. Theory and experiment agree that only for a limited set of combinations of magnetic particle loading and film thickness can both significant bending and shape recovery be achieved. For NdFeB microparticles dispersed in a DiAPLEX matrix, the optimum actuation performance is achieved for a film with a thickness of 60 µm at 40 wt% NdFeB loading. While these parameters are highly relevant to a host of applications, including shape‐programmable soft materials and soft robots, the same design principles could be extended to stiffer shape memory polymers or different sample sizes for deep‐sea or aerospace applications. The future use of untethered sensors will further support applications in remote locations. Furthermore, additive manufacturing of the actuators could allow direct integration of sensors as an intrinsic part of actuator structures at the nexus of soft actuators and soft electronics.

## 3 Experimental Section

### Multifunctional Photothermally and Magnetically Driven Soft Actuators

For combined photothermal and magnetic actuation, NdFeB microparticles (MQP‐15‐7, Magnequench) with an average particle diameter of 5 µm were embedded in shape‐memory polymer films of DiAPLEX (MM5520, SMP Technologies). The loading of NdFeB in the composite was tuned between 10 and 80 wt%. DiAPLEX dissolved in tetrahydrofuran (THF EMPLURA, Sigma Aldrich) via vigorous stirring was mixed with NdFeB microparticles using a vortexer. The homogeneous mixture was poured into a poly(tetrafluoroethylene) (PTFE) mold (length: 50 mm, width: 50 mm), and the THF gradually evaporated at room temperature over 1 h. After evaporation of THF on a hot plate at 70 °C for 1 h, the composite was cooled down to room temperature and a homogeneous 20 kOe magnetic field was applied perpendicular to the surface of the film. To complete the removal of any residual THF, the composite films were annealed in a vacuum oven at 80 °C for 1 h. The film thickness was controlled by monitoring the volume of the mixture added to the mold. The relationship between thickness and volume was established by imaging the cross‐sectional areas of the actuators under a tabletop scanning electron microscope (Phenom XL, Thermo Fisher). For the thickness characterization experiments, sample volumes were varied between 1 and 16 mL in a 30 × 20 mm^2^ areal PTFE mold, yielding actuator thicknesses between 25 and 300 µm.

Sedimentation caused the microparticles to settle to the bottom of the sample, yet for actuators thinner than 100 µm, this effect is not critical. Furthermore, actuation of the origami and the derived theoretical model are not affected by this microparticle distribution, as they are mainly driven by the net magnetic moment of the composite.

Although there are some heterogeneities in the distribution of magnetic microparticles on short length scales (Figures S1, Supporting Information), at a larger length scale relevant to magnetic actuation, the heterogeneities are averaged out, and the distribution is uniform. The local heterogeneities are evident particularly for very thin actuators when the thickness of the actuator becomes comparable to the size of magnetic microparticles. In this case, small local concentration variations can lead to the formation of a patchy pattern observed in the optical micrographs of the actuator (Figure 1). This would be expected to result in spatial variations of the magnetic and mechanical properties, but these variations do not have an observable effect on the actuation behavior. Indeed, the experimental data, even for thin actuators, can be quantitatively reproduced with the theoretical analysis assuming a homogeneous distribution of the mechanical and magnetic properties in the actuator (Figure 1b,c).

The measurements indicate that even under long illumination times of 45 s, photothermal heating of the composite can raise the temperature of the actuator to a maximum of 71 °C (Figure S7, Supporting Information). This temperature is well above the glass transition temperature of the composite^[^ ^35^ ^]^ but low enough to avoid thermal degradation. While no effects of degradation of the composite were seen by using a lamp that includes UV wavelengths (350 nm), for future experiments, this short wavelength could be removed with a longpass filter. Furthermore, the brief illumination times below 5 s may also explain why degradation was not observed.

### Compliant Magnetic Field Sensors

Two types of magnetic field sensors are used in this work. Sensors relying on giant magnetoresistance (GMR)^[^ ^38^ ^]^ arranged in Wheatstone bridges and the anomalous Hall effect^[^ ^39^ ^]^ were prepared for the detection of in‐plane and out‐of‐plane magnetic fields. For preparing magnetosensitive e‐skins, shapeable magnetoelectronic devices^[^ ^40^ ^]^ were fabricated on ultrathin foils.^[^ ^41^, ^42^, ^43^, ^44^ ^]^ Silicon wafers (Crystec, Germany) were used as supporting substrates, which were laminated with 50 × 50 mm^2^ pieces of ultrathin 3 µm‐thick Mylar foils (Chemplex Industries Inc., USA). Water droplets were added between wafers and foils to promote adhesion by capillary forces. Compressed air was used to remove excess water and flatten the foils, which were then spin‐coated at 4000 rpm with AZ 5214E photoresist (MicroChemicals GmbH, Germany) and dried for 5 min at 90 °C. Subsequently, the samples were exposed with a DWL 66 laser writer (Heidelberg Instruments, Germany) to define the sensor patterns, post‐baked on a hot plate at 120 °C for 2 min and flood exposed for 30 s with a UV light source (proMa 140 017, Germany). Next, the samples were developed in AZ 351B solution (MicroChemicals GmbH, Germany) for 60 s, rinsed in deionized water and dried with compressed air. All sensors were laminated to the underlying DiAPLEX‐based composite using a 10 wt% solution of poly(vinyl alcohol) (PVA MW 115 000 ≥ 88%, hydrolyzed, VWR, Germany), which was dried on a hot plate at 70 °C for 10 min.

For the GMR sensors, multilayered stacks of [Ni_81_Fe_19_(1.5 nm)/Cu(2.3 nm)]_30_ coupled at the second antiferromagnetic maximum were deposited on the patterned substrates by magnetron sputtering at room temperature (sputter gas (Ar) pressure: 10^–3^ mbar; base pressure: 10^–7^ mbar; deposition rate: 2 Å s^−1^). To conclude photolithography, the samples were lifted off in acetone, rinsed in ethanol and blow dried. The resulting meander‐shaped sensors had a stripe width of 50 µm. In a second processing step, a metal stack of Ta(2 nm)/Cu(100 nm)/Pt(2 nm) was deposited using magnetron sputtering at room temperature (sputter gas (Ar) pressure: 10^–3^ mbar; base pressure: 10^–7^ mbar; deposition rate: 2 Å s^−1^) to define electrical contacts to the sensors. The width of the contact lines is 800 µm. The GMR effect is quantified based on the analysis of the field dependent resistance, *R*(*H*) with respect to the resistance of the sensor in magnetic saturation, *R*(*H*_sat_): GMR = (*R*(*H*) − *R*(*H*_sat_))/(*R*(*H*_sat_)). When arranged in a Wheatstone bridge, the four GMR sensors comprise an angle sensor of the orientation of the in‐plane magnetic field with respect to the bridge axis. In this work, a full Wheatstone bridge configuration was used. Each of the four magnetic field sensors composing the bridge is nominally the same and equally sensitive to the magnetic field. As the origami structure is rotated in steps of 90°, the resistor closest to the fixed permanent magnet changes, which is predominantly affected by the stray magnetic field of the magnet (Figure S11, Supporting Information). For each orientation, the bridge is differently unbalanced, making it possible to determine the position of the magnet from the output voltage. The resistors on the lower half of the bridge (*R*_3_, *R*_4_) have a larger influence on the output voltage. They constitute the main component of the voltage divider defining *V*_out_, as they are present in both numerator and denominator of the bridge equation: $V_{\text{out}}\;\;=\;\;\left(\frac{R_{4}}{R_{1}\;\;+\;\;R_{4}}\;\;-\;\;\frac{R_{3}}{R_{2}\;\;+\;\;R_{3}}\right)\;\;V_{\text{supply}}$. Therefore, the bridge is more responsive to changes in these resistors. The resistors on the upper half of the bridge (*R*_1_, *R*_2_) do not modify the output to the same extent and thus define two states with lower output voltage variation. The sensor bridge was stimulated by magnetic fields with in‐plane and out‐of‐plane components ≤500 Oe, which allowed it to operate in the linear detection regime (Figure S8, Supporting Information).

It was noted that when using the GMR sensor as an out‐of‐plane sensor, there was an intrinsic asymmetry, which allowed determination of the magnetization state for magnetic fields below 0.5 kOe (Figure S8, Supporting Information). This asymmetry meant that the GMR response did not have the same magnitude for positive and negative magnetic fields. Still, the sensor detected the magnetization state, thus providing the information whether the origami is up, down, or non‐magnetized.

Anomalous Hall effect sensors were prepared by magnetron sputtering of a metal stack of Ta(1 nm)/Co(2 nm)/Pt(1 nm) at room temperature (sputter gas (Ar) pressure: 10^–3^ mbar; base pressure: 10^–7^ mbar; deposition rate: 2 Å s^−1^) on the Mylar foils. The Hall cross layout was defined by photolithography. The Hall cross area is 2 × 2 mm^2^ with a line width of 400 µm. The anomalous Hall effect sensors were electrically contacted using the same layer stack as described above for the GMR sensors.

### Rotation Control for Self‐Guided Assembly

The magnetic origami actuators with a four‐arm configuration were placed on a motorized rotation stage driven by a stepper motor (PO1207, Eckstein, Germany), controlled by a computer running LabVIEW 2015. The stage was designed to ensure the magnetic field from the motor did not directly affect the magnetic origami. A high intensity light source (OSRAM HXP‐R120 W 45C VIS, 2800 lm, integrated 350–720 nm bandpass filter, Leistungselektronik JENA GmbH, Germany) was focused on one of the arms of the actuator to soften it, while the other arms and the central area were not illuminated. A cylindrical NdFeB permanent magnet (15 mm diameter × 8 mm height, S‐15‐08‐N, Supermagnete, Germany) was located close to the illuminated region, providing the magnetic field intensity (≈500 Oe) and gradient necessary to initiate 90° bending of the illuminated arm (Movie S3, Supporting Information). The magnetic field sensor (GMR‐based angle sensor) was laminated on the center of the actuator to detect the location of the permanent magnet. The sensors were biased with a constant voltage of 5 V, and their output voltage was measured differentially with a USB‐6211 data acquisition device (National Instruments, USA). The angle of the magnet with respect to the sensor axis was measured from the voltage of the GMR sensor, for which the response curve was established (Figure 2e). Using this feedback, the LabVIEW program can precisely determine the position of the stage with respect to the magnet and trigger the next rotation step. Successive guided lifting and rotation steps result in the lifting of all four arms. During each lifting step, the light was first switched on to trigger the fold. As soon as folding was completed, the light was switched off to fix the position of the arm.

### Electromagnet‐Driven Sequential Folding of an Extended Film

A magnetic origami actuator in the shape of a boat‐like structure was prepared from 40 × 40 mm^2^ sheets by cutting off two triangles from the upper corners. The resulting shape was capped with a 3 µm‐thick sensor foil hosting four Hall effect sensors distributed over the top, center, and lower right and left ends of the actuator. The sensors were connected in series and supplied with a custom‐made constant current source set to deliver 1 mA. The voltage outputs of the sensors were connected with 50 µm copper wires threaded under the robot and interfaced with a board to the analog inputs of a USB‐6211 data acquisition device. The actuators equipped with sensors were attached on a white paper stage fixed above an electromagnet (ITS‐MS‐7040‐12VDC, Intertec Components, Germany). The polarity of the power supply for the electromagnet was adjusted to ensure the ends of the actuator were attracted toward the center. Illuminating specific ends of the actuator allowed folding in a controlled sequence, thus establishing sequences 1 (top, right, then left) and 2 (right, left, then top). The entire process was monitored electronically with LabVIEW (Movie S5, Supporting Information).

Resistive magnetic field sensors are used in this study. Therefore, they are sensitive not only to the magnetic field but also to variations in temperature caused by heating when the sample is illuminated. The typical time trace of the sensor signal is shown in Figure S12, Supporting Information. The sensor registers a clear signal change when the magnetic field is switched on or off. However, the thermal effects of heating from illumination cause the signal to drift. According to the data shown in Figure S7, Supporting Information, the temperature increases gradually, since several seconds are needed to observe a noticeable temperature change. In contrast, the magnetic field is switched on much faster, on the ms time scale. This difference in the timing of both effects allows for their easy discrimination. For instance, to monitor the effect of the magnetic field, the signal immediately after switching the magnetic field should be analyzed. All other data should be disregarded as unrelated to magnetic‐field‐induced changes. Following this argument, only the relevant data related to the magnetic‐field‐induced changes in the sensor signal is presented in Figure 3. By analyzing the steady states of the actuator, the actual variation due to the movement from the initial (flat) to the final (bent) position can be determined. Although it is possible to distinguish the orientation of the sensor from the effects of temperature by relying on differences in the time scales of actuation and thermalization, temperature changes could be more generally compensated by adding temperature sensors to the origami actuators. The signal arising from changes in orientation could then be calculated from the more complex signal.

### Mapping the Magnetic Stray Fields of an Electromagnet and a Magnetic Composite

The bending performance of the magnetic composite (Figure 1c) was investigated using an electromagnet (ITS‐MS‐3025, Intertec Components, Germany) biased at 12 VDC. The stray magnetic field components of the electromagnet were measured with a Gaussmeter (HGM09s, MAGSYS, Germany) mounted in a micromanipulator, which allowed measurements of the in‐plane and out‐of‐plane components of the field in 0.5 mm steps. The center of the electromagnet upper face was defined as the origin of the cylindrical coordinate system. The probe of the Gaussmeter was swept along the radial direction while keeping the distance from the face of the magnet constant, which was repeated at several distances, 0, 2, 4, 8, and 12 mm. The radial component *H*_r_ of the field was measured at each point with the Hall cross perpendicular to the upper face of the electromagnet, while the *z*‐component *H*_*z*_ was obtained by pointing the probe parallel to the top face of the electromagnet (Figure S5, Supporting Information).

The stray field produced by the origami actuator after magnetization (Figure 1h) was measured following the same procedure described above. The Hall probe, mounted in a micromanipulator, was swept perpendicular to the actuator surface in 20 µm steps until the measured stray field fell below the geomagnetic field. The orientation of the Hall probe was maintained parallel to the plane of the film during this experiment.

### Modeling Magneto‐Origami Actuators

A film possessing a magnetic moment **m** will experience a torque in the presence of a magnetic flux density **B** given by ***τ***_M_ = **m** × **B**, where **B** is proportional to the magnetic field **H** in free space, **B** = *µ*_0_ **H**. In addition, a non‐uniform magnetic field may also exert forces on the film according to **F** = ∇(**m**·**B**). These forces may result in torques about the hinge that are independent of the field‐mediated torques in the former expression. A detailed analysis of each of these torques calculated for the experimentally determined magnetic stray field landscape above the electromagnet is summarized in the next section. It is clear from this analysis that, while gradient‐mediated torques modify the actuation behavior in limited regions, the phenomenology of the electromagnet system is dominated by field‐mediated magnetic torques, which are strongly predictive of the gross behavior of the film. Thus, for qualitative analysis of this system, it is sufficient to consider only field‐mediated torques in modeling actuation. While this simplified model is limited in its ability to precisely predict the details of folding, it remains a powerful framework for understanding the behavior of the system, including dependencies on key system parameters—film thickness, magnetic field geometry, magnetic particle loading, and polymer modulus.

### Balance of Magnetic and Elastic Torques

A model is developed for a magnetic film of thickness *a* folded through an angle ϕ by a magnetic field (Figure S3a, Supporting Information). It is assumed that some length of the film *L*_E_ is bent through a constant curvature, while a length of film *L*_M_ is predominantly responsible for magnetic torques. As discussed above, the torque due to the magnetic field is dominated by ***τ***_M_ = **m** × **B**. As a first‐order approximation, the directional dependence was neglected and only the maximum value of the product was considered, or τ_M_ = *MwaL*_M_ *B*, where *M* is the magnetization of the film, *w* is the width of the film, and thus *waL*_M_ is the volume of the section of the magnetic film involved in actuation. The elastic energy of the curved segment of the film, *L*_E_, is approximated with the Kirchhoff model.^[^ ^45^ ^]^ For a uniform elastic rod bent to a constant radius of curvature, this elastic energy reduces to

(2) $$\begin{array}{c} U_{E}\;\;=\;\;\frac{1}{2}\;EI\;\;\frac{\phi^{2}}{L_{E}} \end{array}$$

where *I* is the second moment of area and *E* is the modulus of the film. Thus, the magnitude of elastic torque on the film is given by

(3) $$\begin{array}{c} \tau_{E}=\frac{dU_{E}}{d\theta }=2\frac{dU_{E}}{d\phi }=2EI\frac{\phi }{L_{E}} \end{array}$$

where the angle θ = ϕ/2 is defined in Figure S3a, Supporting Information. The film is expected to rest at equilibrium when τ_E_ = τ_M_, for which the bending angle is

(4) $$\begin{array}{c} \phi \;\;=\;\;MwaL_{E}\;L_{M}B/\left(2EI\right) \end{array}$$

Finally, for a film of rectangular cross section, the second area moment is given by $I=\frac{1}{12}\;\;wa^{3}$, yielding a predicted bending angle of

(5) $$\begin{array}{c} \phi \;\;=\;\;6\frac{MB}{Ea^{2}}\;\;L_{E}L_{M} \end{array}$$

If the radius of the bend *R* is important in the design of a device, we may modify Equation (5) with the substitution *L*_E_ = *R*ϕ (Figure S3a, Supporting Information). Subsequently solving for *R* yields an expression for the radius of curvature, which is independent of *L*_E_:

(6) $$R=\frac{1}{6}\;\frac{Ea^{2}}{MBL_{M}}$$

Here, it was seen that lower bending rigidity, lower thickness and greater magnetic torques will reduce the radius of the bend. It is important to note in Figure S3a, Supporting Information that the two occurrences of ϕ are equal and that *L*_E_ is the arc length subtended by ϕ.

### Influence of Magnetic Particle Loading

In Equation 5, both the magnetization *M* and the modulus *E* depend on the loading of magnetic particles within the polymer. The magnetization can be calculated, *M*(*f_v_*) = *M*_NdFeB_ *f_v_*, where *M*_NdFeB_ is the magnetization of the NdFeB particles and *f*_v_ is the volume fraction of magnetic particles in the composite. The loading‐dependent modulus may be best described by inverse rule of mixtures,^[^ ^46^ ^]^ which suggests that the modulus of a composite is given by,

(7) $$\begin{array}{c} E_{c}\;\;=\;\;{\left(\frac{f_{v}}{E_{f}}\;\;+\;\;\frac{1\;\;-\;\;f_{v}}{E_{m}}\right)}^{-1} \end{array}$$

where *E*_c_, *E*_f_, and *E*_m_ are the Young's moduli of the composite, filler (NdFeB particles), and DiAPLEX matrix, respectively. In the case of *E*_f_ ≫ *E*_m_, which is certainly satisfied in the system (the modulus of NdFeB particles is several orders of magnitude larger than that of the DiAPLEX matrix), Equation 7 reduces to $E_{c}\;\;\approx \;\;\frac{E_{m}}{\left(1\;\;-f_{v}\right)}$. With these approximations, the expression for the bending angle becomes

(8) $$\begin{array}{c} \phi \;\;=\;\;6\frac{M_{\text{NdFeB}}\;f_{v}\left(1\;\;-\;\;f_{v}\right)}{E_{D}a^{2}}\;BL_{E}L_{M} \end{array}$$

The volume fraction, *f*_v_, may be written in terms of weight fraction *f*_w_ using the following expression, where ρ_D_ and ρ_NdFeB_ are the mass densities of DiAPLEX and NdFeB, respectively:

(9) $$\begin{array}{c} f_{v}\;\;=\;\;\frac{f_{w}\rho_{D}}{f_{w}\left(\rho_{D}\;\;-\;\;\rho_{\text{NdFeB}}\right)\;\;+\;\;\rho_{\text{NdFeB}}} \end{array}$$

### Temperature Dependence of the DiAPLEX Modulus

It should be noted that the modulus of unloaded DiAPLEX depends on temperature:

(10) $$\begin{array}{c} E_{\text{Diaplex}}\left(T\right)\;\;=\;\;E_{0}\left(1\;\;-\;\;\beta \tan h\;\;\left[\frac{\left(T\;\;-\;\;T_{0}\right)}{T_{C}}\right]\;\right)\;\;\text{​}\text{MPa} \end{array}$$

Over the temperature range 20 °C ≤ *T* ≤ 80 °C, the parameters of Equation 10 are *E*_0_ = 468 MPa, β = 0.98, *T*_C_ = 7.4 °C, and *T*_0_ = 35 °C.^[^ ^29^ ^]^ Figure 1b then shows the predicted bending angle as a function of film thickness and magnetic particle loading for a film with *M*_NdFeB_ = 8 × 10^5^ A m^−1^, ρ_NdFeB_ = 7600 kg m^−3^, ρ_D_ = 1200 kg m^−3^, *B* = 10 mT (*H* = 7960 A m^−1^ or 100 Oe), *L*_M_ = *L*_E_ = 1 cm, and *E*_D_ = 45 MPa @ 46.9 °C. An external magnetic field of 100 Oe was used since it was typical of the space above the magnet during experiments.

### Calculating Magnetic Torques

A thin film with a fixed uniform magnetization *M* resulting in a net magnetic moment **m** perpendicular to the plane of the film bends at a hinge location *r*_h_ through an angle ϕ, as depicted in Figure S3a, Supporting Information. Bending is restricted to the *rz*‐plane.

### Field‐Induced Torques

An applied magnetic field will create a torque on the moment according to ***τ*** = **m** × **B**. The magnetic torque on an infinitesimal volume element of the film, *d* **τ**_F_ = *d* **m** × **B**, becomes

(11) $$\begin{array}{c} d\tau_{F}\left(r,z\right)\;\;=\;\;\pm dm\left[B_{z}\left(r,z\right)\sin\phi \;\;+\;\;B_{r}\left(r,z\right)\cos\phi \right]\hat{y} \end{array}$$

where $\hat{y}$ is out of the page and the sign depends on the direction of *d* **m**, with the positive corresponding with the direction of *d* **m** indicated in the figure. In this expression, *dm* = |*d* **m**| = *M* *d* *V* is the product of the magnetization with an infinitessimal volume element. Recognizing that

(12) $$\begin{array}{c} \cos\phi \;\;=\;\;\frac{\left(r-r_{h}\right)}{\sqrt{{\left(r-r_{h}\right)}^{2}+z^{2}}}\text{​}\;\;\text{and}\;\;\text{​}\sin\phi \;\;=\;\;\frac{z}{\sqrt{{\left(r\;\;-\;\;r_{h}\right)}^{2}\;\;+\;\;z^{2}}} \end{array}$$

It is seen that the torque is

(13) $$\begin{array}{c} d\tau_{F}\left(r,z\right)\;\;=\;\;\pm \frac{dm}{\sqrt{{\left(r\;\;-\;\;r_{h}\right)}^{2}+z^{2}}}\left[zB_{z}\left(r,z\right)\;\;+\;\;\left(r\;\;-\;\;r_{h}\right)B_{r}\left(r,z\right)\right]\hat{y} \end{array}$$

Thus, dividing the expression above by *dm* yields the magnetic torque per volume per unit magnetization (N m^–1^A^–1^, or T):

(14) $$\begin{array}{c} {\tau^{\prime }}_{F}\left(r,z\right)\text{​}\;\;=\;\;\frac{d\tau_{F}\left(r,z\right)}{dm}\;\;=\;\;\pm \;\;\frac{1}{\sqrt{{\left(r-r_{h}\right)}^{2}+z^{2}}}\;\;\left[zB_{z}\left(r,z\right)\;\;+\;\;\left(r\;\;-\;\;r_{h}\right)B_{r}\left(r,z\right)\right]\;\hat{y} \end{array}$$

This term provides a map of the ability of a given magnetic field to exert torque in all space, independent of the magnitude of the magnetization of the film. For the electromagnet used in this work (Figure S5, Supporting Information), this calculated map is shown in the top row of Figure S3b, Supporting Information.

### Gradient‐Induced Torques

A non‐uniform magnetic field will also exert forces on the film according to **F** = ∇(**m**·**B**), and these will result in torques about the hinge, which are independent of the field‐mediated torques in the previous section. The magnetic force on an infinitesimal volume element of the film *d* **F** = ∇(*d* **m**·**B**) thus becomes

(15) $$\begin{array}{l} dF\;\;=\;\;\pm \frac{\partial }{\partial r}\;\;\left[dmB_{r}\sin\phi +dmB_{z}\cos\phi \right]\hat{r}\\ \text{​}\text{​}\text{​}\text{​}\text{​}\text{​}\text{​}\text{​}\text{​}\text{​}\text{​}\text{​}\text{​}\text{​}\text{​}\;\;\;\;\;\;\;\;\text{​}\pm \frac{\partial }{\partial z}\;\;\left[dmB_{r}\sin\phi \;\;+\;\;dmB_{z}\cos\phi \right]\hat{z} \end{array}$$

For a uniform magnetic moment, this reduces to

(16) $$\begin{array}{l} dF\;\;=\;\;\pm dm\left[\sin\phi \frac{\partial B_{r}}{\partial r}\;\;+\;\;\cos\phi \frac{\partial B_{z}}{\partial r}\right]\hat{r}\\ \text{​}\text{​}\text{​}\text{​}\text{​}\text{​}\text{​}\text{​}\text{​}\text{​}\text{​}\text{​}\;\;\;\;\;\;\;\;\text{​}\pm \;dm\left[\sin\phi \frac{\partial B_{r}}{\partial z}\;\;+\;\;\cos\phi \frac{\partial B_{z}}{\partial z}\right]\hat{z} \end{array}$$

or

(17) $$\begin{array}{c} dF=\pm \frac{dm}{\sqrt{{\left(r-r_{h}\right)}^{2}+z^{2}}}\left[\left(z\frac{\partial B_{r}}{\partial r}+\left(r-r_{h}\right)\frac{\partial B_{z}}{\partial r}\right)\hat{r}+\left(z\frac{\partial B_{r}}{\partial z}+\left(r-r_{h}\right)\frac{\partial B_{z}}{\partial z}\right)\hat{z}\right] \end{array}$$

Forces exerted at a distance from the hinge point will result in a torque according to **τ** = **r** × **F**, where **r** is the location of the force. Thus, the gradient‐mediated magnetic force above will result in a torque per volume on the film given by $d\tau_{G}\;\;=\;\;(rdF_{z}\;\;-\;\;zdF_{r})\;\hat{y}$, or, dividing by *dm*, a torque per volume per unit magnetization (N m^–1 ^A^–1^, or T) of:

(18) $$\begin{array}{l} d\tau_{G}^{\prime }=\frac{d\tau_{G}\left(r,z\right)}{dm}\pm \frac{1}{\sqrt{{\left(r-r_{h}\right)}^{2}+z^{2}}}[\left(r-r_{h}\right)\left(z\frac{\partial B_{r}}{\partial r}+\left(r-r_{h}\right)\frac{\partial B_{z}}{\partial r}\right)-z\left(z\frac{\partial B_{r}}{\partial z}+\left(r-r_{h}\right)\frac{\partial B_{z}}{\partial z}\right)]\hat{y} \end{array}$$

This term provides a map of the ability of a given magnetic field gradient to exert torque in all space, independent of the magnitude of the magnetization of the film. For the electromagnet used in this work (Figure S5, Supporting Information), this map is shown in the middle row of Figure S3b, Supporting Information.

The sum of field torques and gradient torques yields the net magnetic torque on the film, as shown in the bottom row of Figure S3b, Supporting Information, and this ultimately suggests the behavior of a film in this space. Notably, a film with a hinge set at a radial distance of 10 mm from the magnet axis (Figure S3b, middle column, Supporting Information) will experience a uniform counter‐clockwise torque from the magnetic field (Figure S3b, top row, Supporting Information) with the exception of a small region of clockwise torque just to the left of the hinge. This region will limit the closure of a folding film; indeed, in the absence of other influences a film would come to rest on the zero‐torque contour indicated by the black line. This effect is diminished by shifting the hinge point toward the magnetic axis (8 mm; Figure S3b, left column, Supporting Information) and exacerbated by shifting in the opposite direction (12 mm; Figure S3b, right column, Supporting Information).

Torques due to the magnetic field gradient (Figure S3b, middle row, Supporting Information) are generally consistent with an attractive (downward) force. This promotes closure of a fold, minimizing the regions of clockwise torque to the left of the hinge (region B). However, it also provides a barrier to the initiation of a fold (region A), which may affect performance. As the hinge moves away from the magnet axis (12 mm; Figure S3b, right column, Supporting Information), this barrier is lessened.

It is evident from Figure S3b, Supporting Information that both field torques and gradient torques play non‐negligible roles in the actuation of a film. However, it is also clear that the magnetic field torques dominate the phenomenology of the system; that is, field torques alone are strongly predictive of the ultimate behavior of the film (in this case, to fold counter‐clockwise), while gradient torques modify this behavior in limited regions.

## Conflict of Interest

The authors declare no conflict of interest.

## Supporting information

Supporting Information

Supplemental Movie 1

Supplemental Movie 2

Supplemental Movie 3

Supplemental Movie 4

Supplemental Movie 5

Supplemental Movie 6

## Data Availability

Research data are not shared.
