# Supplementary material for: Reconfigurable Magnetic Origami Actuators with On‐Board Sensing for Guided Assembly
Source: Adv Mater. 2021 May 9;33(25):2008751. doi: 10.1002/adma.202008751 (PMC11481057; doi:10.1002/adma.202008751)
Supplement: Supplementary file 1 — Supporting Information [file ADMA-33-2008751-s004.pdf]

© 2021 Wiley-VCH GmbH

ADVANCED MATERIALS

Supporting Information

for *Adv. Mater.,* DOI: 10.1002/adma.202008751

Reconfigurable Magnetic Origami Actuators with On-Board Sensing for Guided Assembly

Minjeong Ha, Gilbert Santiago Cañón Bermúdez, Jessica A.-C. Liu, Eduardo Sergio Oliveros Mata, Emily E. Evans, Joseph B. Tracy, and Denys Makarov**

© 2021 WILEY-VCH GmbH

Supporting Information

Reconfigurable Magnetic Origami Actuators with On−Board Sensing for Guided Assembly

Minjeong Ha, Gilbert Santiago Cañón Bermúdez, Jessica A.-C. Liu, Eduardo Sergio Oliveros Mata, Emily E. Evans, Joseph B. Tracy, Denys Makarov**

Supporting Movies:

Movie S1 (.mp4 format). Magnetization state detection by offset voltage for magnetized and non-magnetized actuators.

Movie S2 (.mp4 format). Comparison of actuation of magnetized and non-magnetized actuators.

Movie S3 (.mp4 format). Self-guided rotational assembly of a magnetic origami.

Movie S4 (.mp4 format). Characterization of distributed sensors on a boat-shaped actuator.

Movie S5 (.mp4 format). Sequential folding of a boat-shaped magnetic origami using an electromagnet.

Movie S6 (.mp4 format). Sequential folding of a boat-shaped magnetic origami using a permanent magnet.

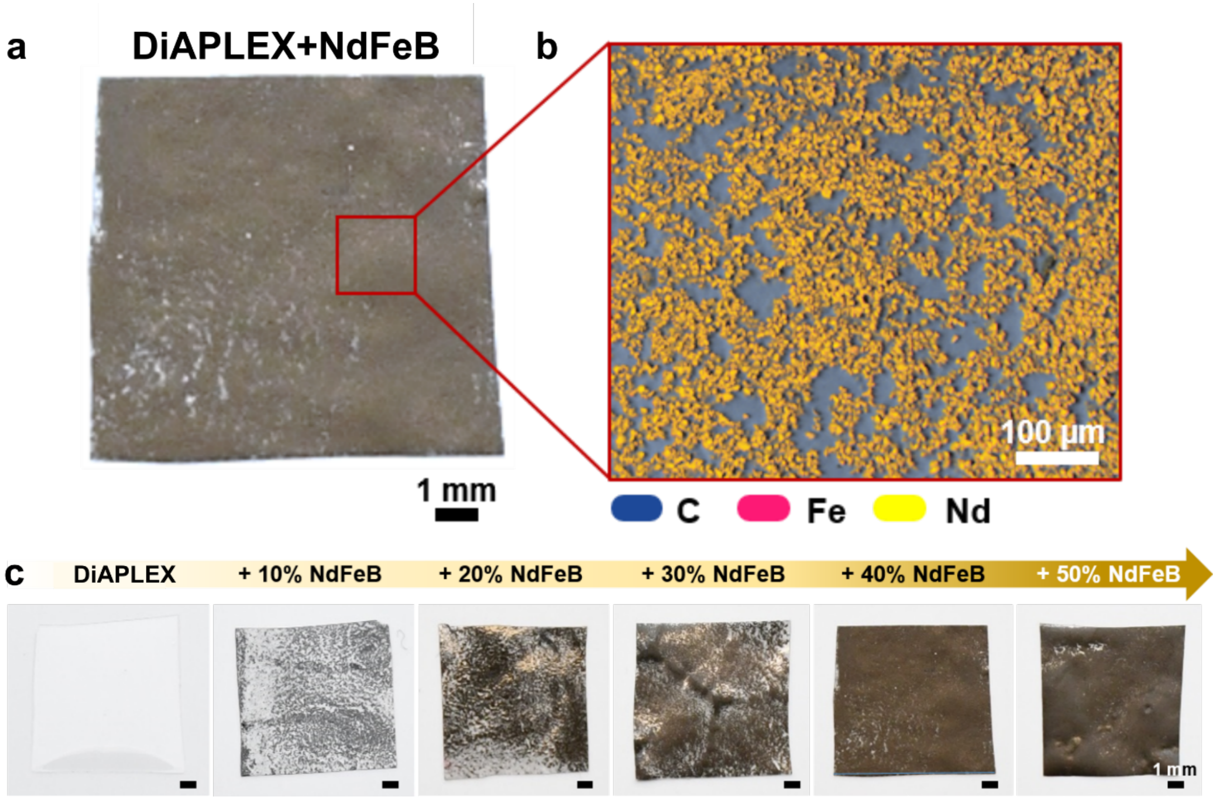

Figure S1. Optical characterization and elemental mapping of NdFeB microparticles embedded in DiAPLEX films. **a**) Photograph and **b**) EDX (in SEM) mapping analysis of randomly dispersed 40 wt% NdFeB microparticles in DiAPLEX films. **c**) Photographs of DiAPLEX films with different loadings of NdFeB microparticles.

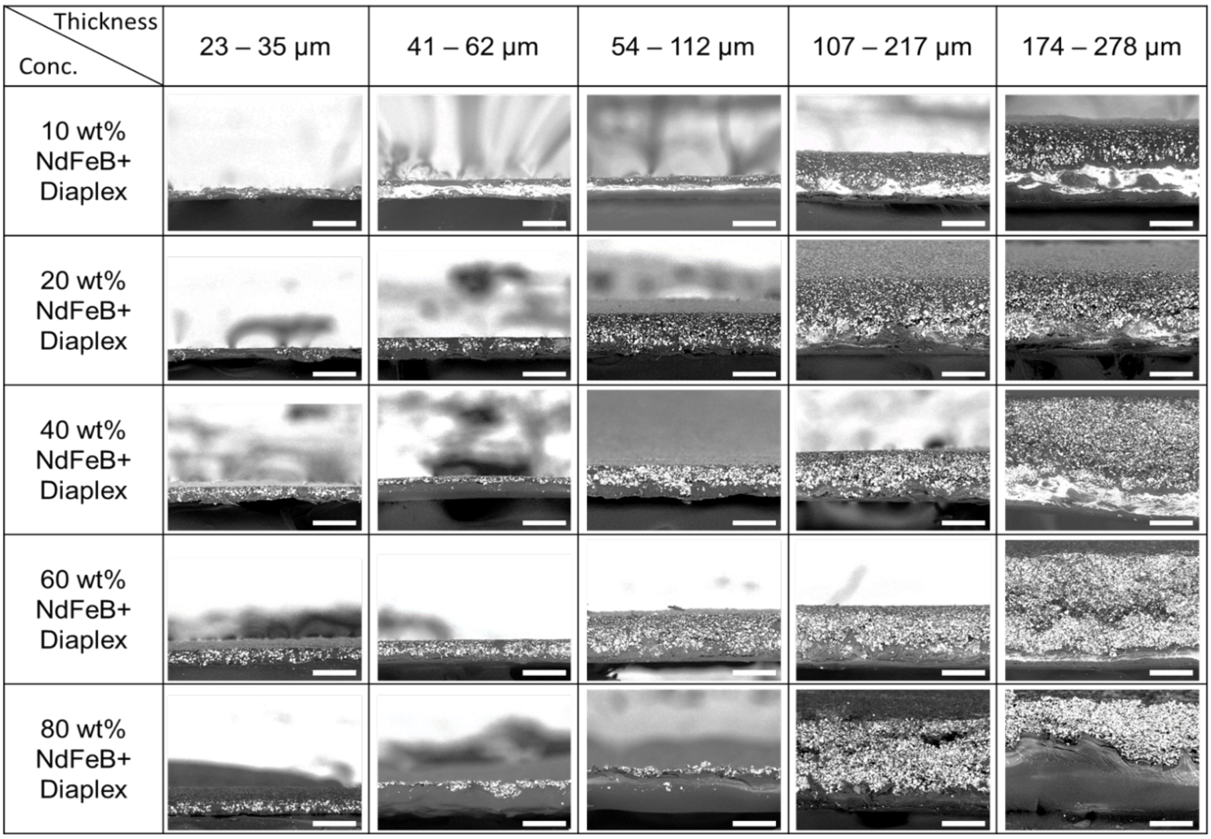

Figure S2. Morphology of DiAPLEX films as a function of thickness. Cross-sectional SEM images of NdFeB microparticles embedded in DiAPLEX films for a range of thicknesses with different loadings of NdFeB microparticles. In each image, the scale bar indicates a length of 100 μm.

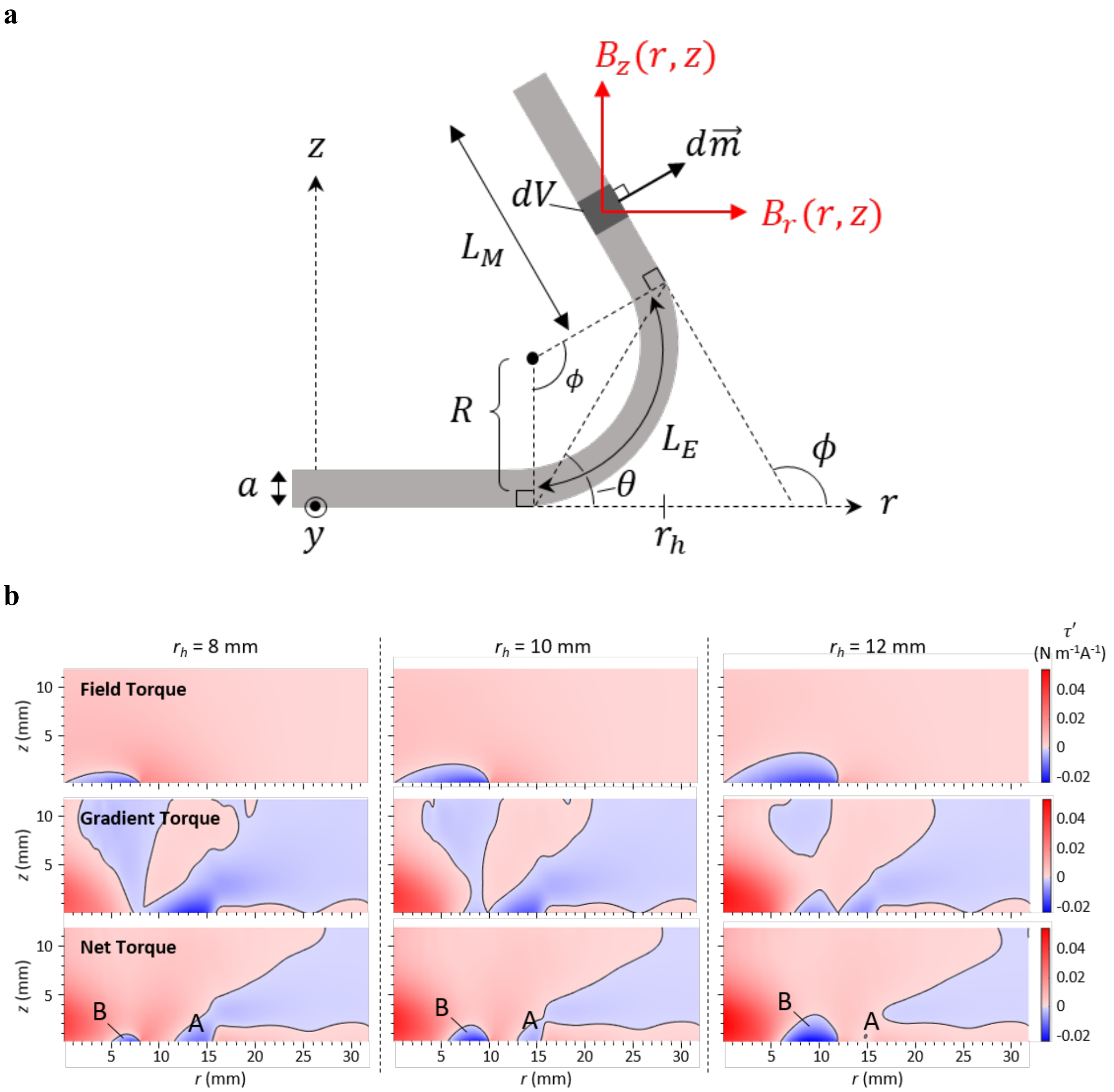

Figure S3. Theoretical insights into the bending process. **a**) Geometry of the bent film for magnetic torque calculations. **b**) Magnetic torques for three different hinge locations. Plots indicate magnetic torque per volume per unit magnetization ($Nm^{-1}A^{-1}$) at a radial distance r from the center of the magnet and height z above the face of the magnet for films folding at hinge locations r_h = 8 mm, r_h = 10 mm, and r_h = 12 mm. Red and blue indicate positive (counterclockwise) and negative (clockwise) torques, respectively. The top row shows that torques due to field alignment (Eq. 14) are counterclockwise throughout most of the space, with a small region of clockwise torques near the magnet center, which resists closure of the fold. In contrast, torques due to the magnetic field gradient (second row, Eq. 18) result from generally attractive (downward) forces near the magnet that cause strong clockwise torques to the right of the fold and strong counterclockwise torques to the left of the fold. Net torque is shown in the bottom row. The phenomenology of actuation in this system is well described by the magnetic field torques (top row), though magnetic field gradients play a non-negligible role in inhibiting the initial folding of an open film and supporting the final folding stage as a film closes. Shifting the location of the fold away from the magnet center generally reduces the initial barrier to folding (A) but limits the ability of the film to close completely (B).

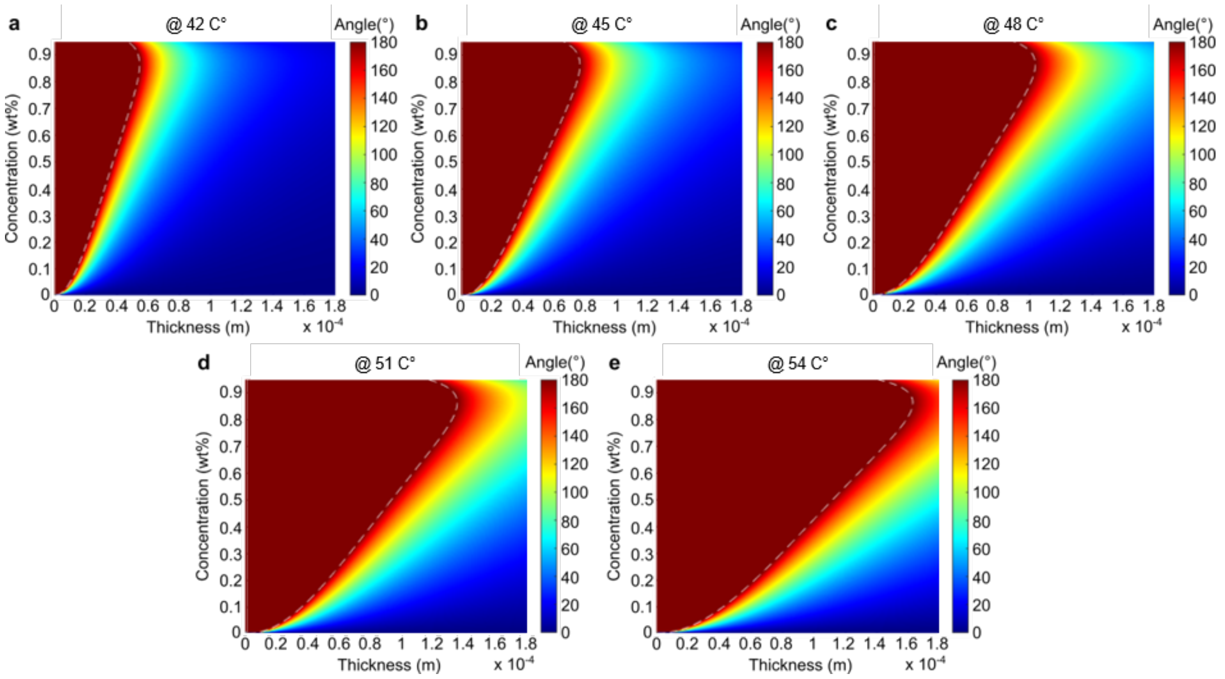

Figure S4. Theoretical predictions of the folding angle as a function of NdFeB loading and thickness at different temperatures. Contour plots depict the parameter range in which a particular folding angle is achieved for temperatures of **a**) 42, **b**) 45, **c**) 48, **d**) 51 and **e**) 54 °C.

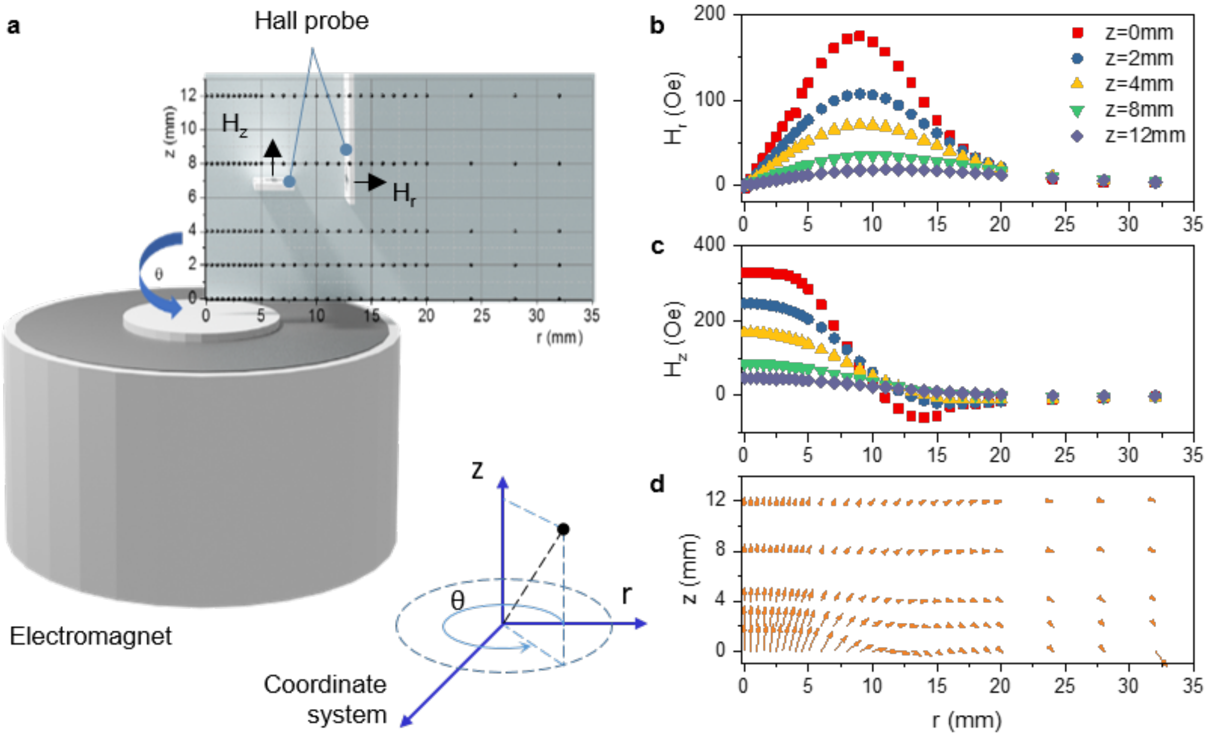

Figure S5. Magnetic field profile of the electromagnet. **a**) Scheme for measuring the magnetic field components of the electromagnet. Dimensions of the electromagnet (outer diameter 63 mm, inner core diameter 28 mm). **b**) Radial H_r and **c**) perpendicular H_z components of the magnetic field produced by the electromagnet (ITS-MS-3025) driven with a current of 0.32 A. Each plot is a fixed distance z from the upper face of the magnet while varying the radial distance r. **d**) Magnetic field vector representation measured on the representative rz-plane.

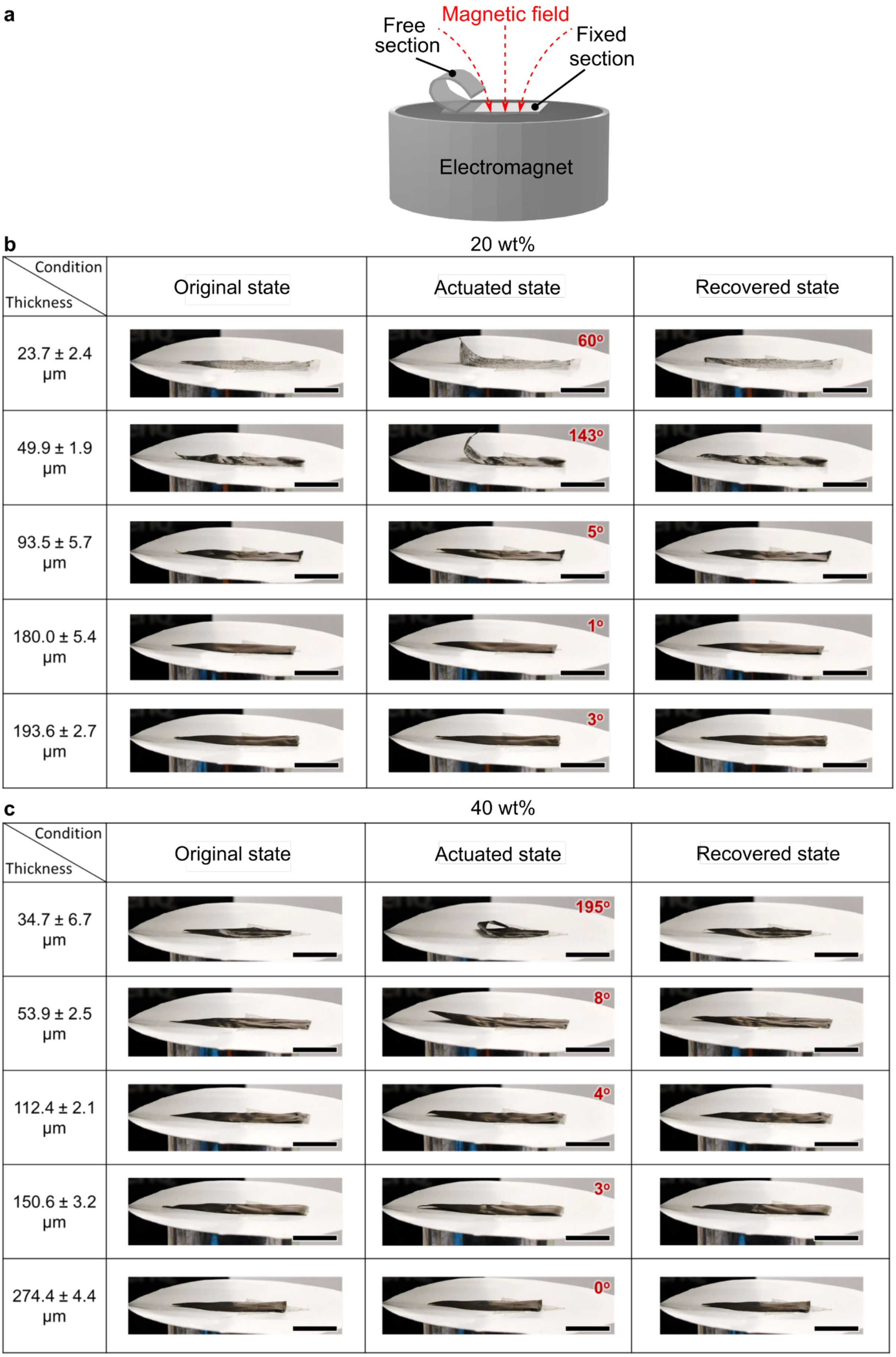
a
Magnetic field
Free section
Fixed section
Electromagnet
b
20 wt%
Condition
Thickness
Original state
Actuated state
Recovered state
23.7 ± 2.4 μm
60°
49.9 ± 1.9 μm
143°
93.5 ± 5.7 μm
5°
180.0 ± 5.4 μm
1°
193.6 ± 2.7 μm
3°
c
40 wt%
Condition
Thickness
Original state
Actuated state
Recovered state
34.7 ± 6.7 μm
195°
53.9 ± 2.5 μm
8°
112.4 ± 2.1 μm
4°
150.6 ± 3.2 μm
3°
274.4 ± 4.4 μm
0°

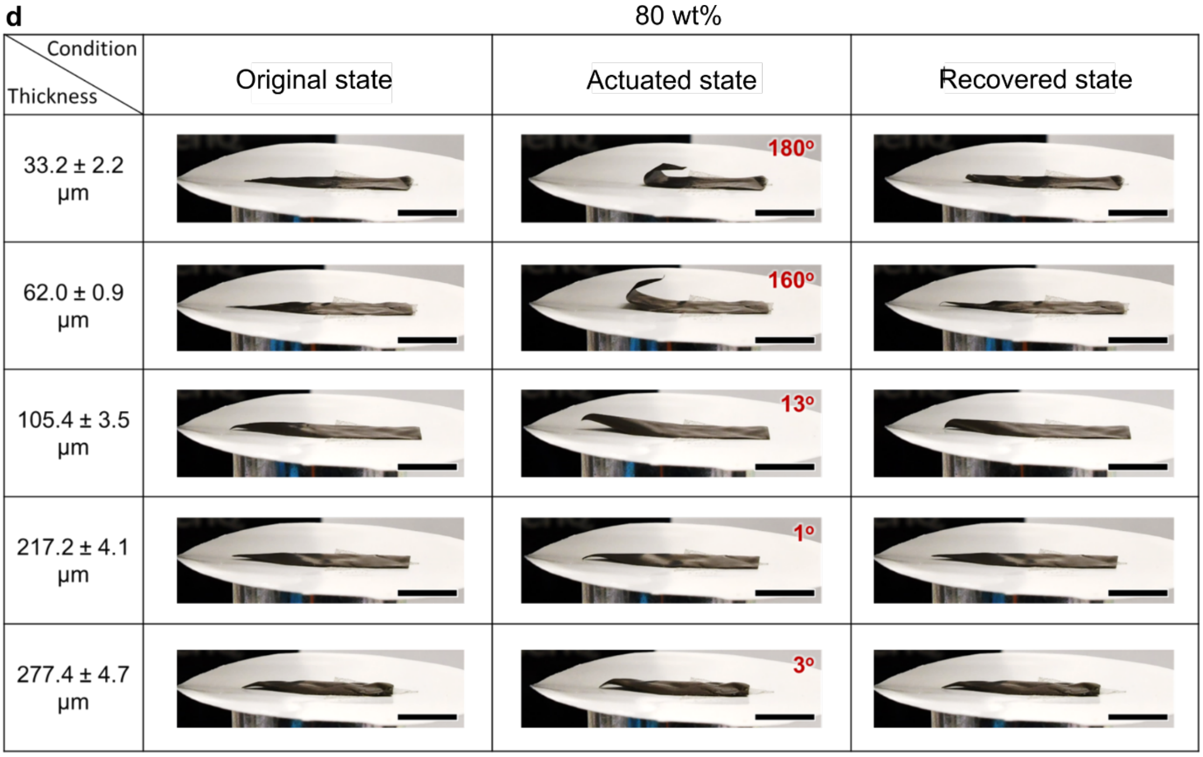

Figure S6. Actuation of composite films for different NdFeB loadings and film thicknesses. **a**) Schematic of the setup used for testing actuation of the magnetic origami. Each sample was illuminated for 5 s while applying a 500 Oe magnetic field produced by the electromagnet described in Figure S5 (Supporting Information) from an applied current of 0.47 A. The magnetic field gradient prompts the free section of the origami to curl towards the center of the electromagnet. Photographs show the actuation response of cantilevers (30 × 20 mm^2) loaded with **b**) 20 wt%, **c**) 40 wt% and **d**) 80 wt% NdFeB microparticles. In each image, the scale bar indicates a length of 1 cm.

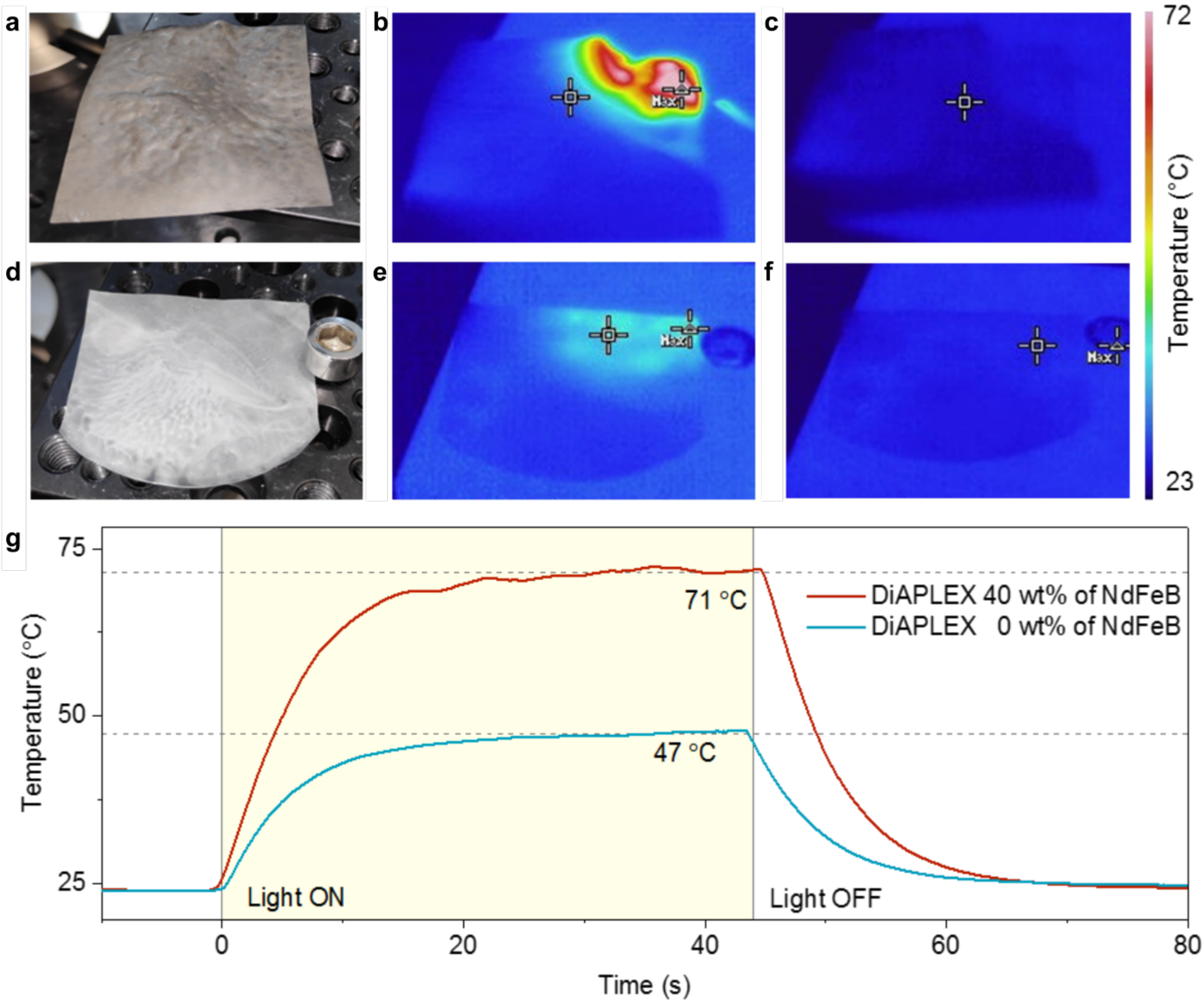

Figure S7. Heating of the composite films with light. **a**) DiAPLEX film with 40 wt% NdFeB microparticles. **b**) Infrared thermography (IRT) showing the temperature upon illumination at one corner and **c**) after cooling to room temperature. **d**) DiAPLEX film without NdFeB microparticles, **e**) IRT showing its temperature upon illumination, and **f**) after cooling down to room temperature. **g**) Temperature curves of both films over time, showing their heating and cooling behaviors. Temperature curves in panel **g**) are measured in the center of the illumination area where the maximum temperature was achieved.

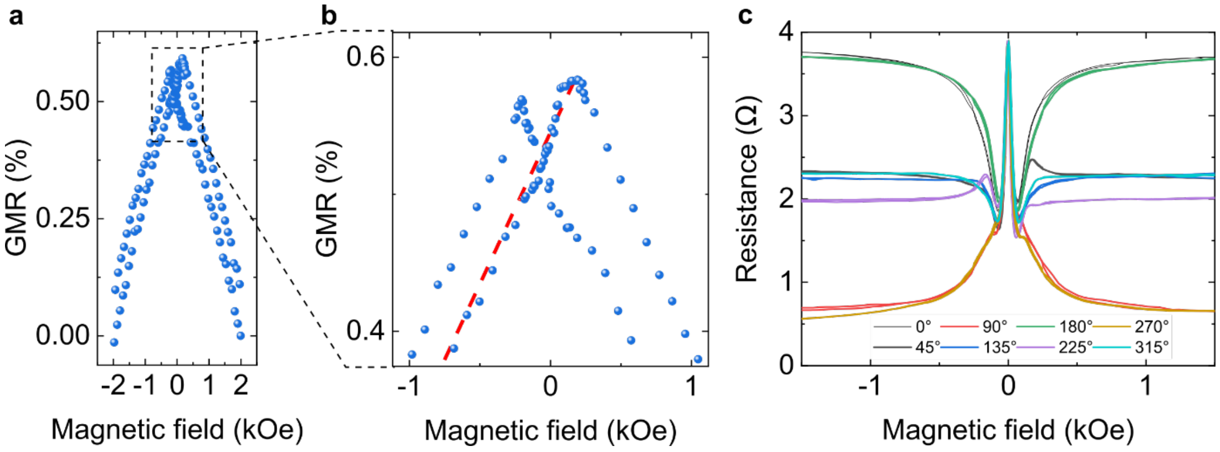

Figure S8. GMR response for compliant magnetic field sensors configured as a Wheatstone bridge. **a**) GMR hysteresis loop measured as a function of the magnetic field in an out-of-plane configuration. Two curves correspond to the measurement in the decreasing magnetic field from maximum positive to maximum negative field and back. **b**) Magnified selection of the data in **a**) highlighting the linear response of the sensor for magnetic fields below ±1 kOe. **c**) Resistance as a function of the in-plane magnetic field applied at different angles.

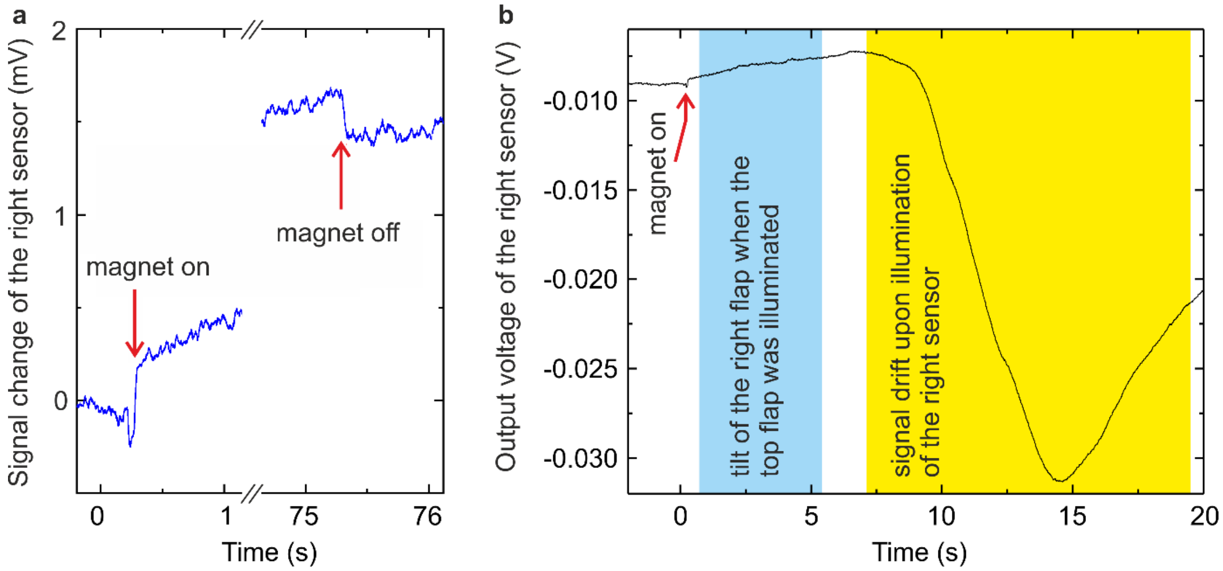

Figure S9. Time evolution of the anomalous Hall effect sensor mounted on the right flap of the actuator shown in Figure 3 of the main text (see also Movie S5, Supporting Information). **a)** The sensor can detect the events when the magnetic field was switched on and off. The signal change due to the magnetic field is in the range of 200 µV and is comparable to the other sensors shown in Figure 3d of the main text. **b)** The sensor detects actuation of the right flap, when the top flap was exposed to light. As the illuminated area is rather broad, the right flap is also partly exposed to light, which leads to its folding. This event can be identified by the gradual increase of the signal after the magnet is on (indicated by the blue shaded region). This tilt is also visible in the Movie S5 (Supporting Information). However, when the right flap was directly illuminated by light, the sensor registered a very strong signal change (indicated by the orange shaded region). The reason for this change is not fully clear. Based on the amplitude of the signal, it is not related to the change in the magnetic field upon actuation of the right flap. After the light was moved away from the right flap, the signal of the right sensor recovers (although with an offset), and the sensor can detect the event, when the magnetic field was switched off (panel **a**)).

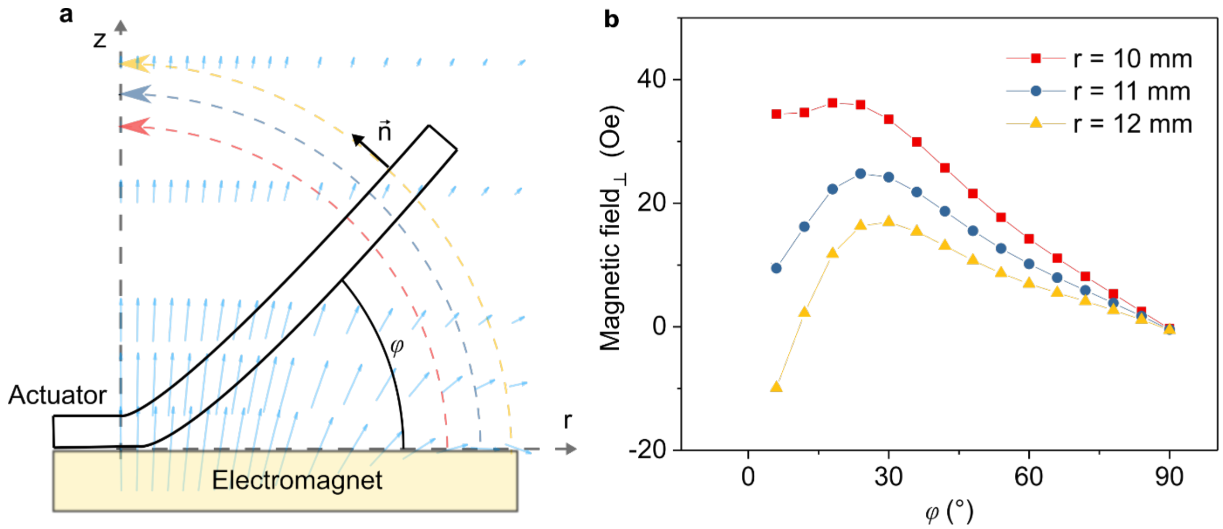

Figure S10. Magnetic field measured by an anomalous Hall effect sensor during folding demonstrated in Figure 3 of the main text (see also Movie S5, Supporting Information). **a)** During the folding process, the actuator travels through the spatially inhomogeneous magnetic field (blue arrows) generated by the electromagnet. The field components are presented in Figure S5 (Supporting Information). The anomalous Hall effect sensor measures the magnetic field applied in the out-of-plane direction. Hence, it senses the magnetic field component perpendicular to the actuator surface $\vec{n}$ during the actuation from $\varphi = 0°$ to 90°. **b)** Calculated magnitude of the magnetic field component normal to the sensor surface during the folding process with respect to the folding angle. Each curve corresponds to a different trajectory defined by circular arcs (schematically shown with dashed curves in panel **a)**) with radius r = 10, 11, or 12 mm. To calculate the normal component of the magnetic field during actuation, which changes the angle of the magnetic field sensor, we interpolate the magnetic field profile produced by the electromagnet (Figure S5, Supporting Information) along the actuation path using a polynomial fit to the experimental data. The curves are non-monotonic with a maximum at ~25°, which explains the presence of the maximum in the time evolution of the voltage signals measured by the anomalous Hall effect sensors in Figure 3d of the main text.

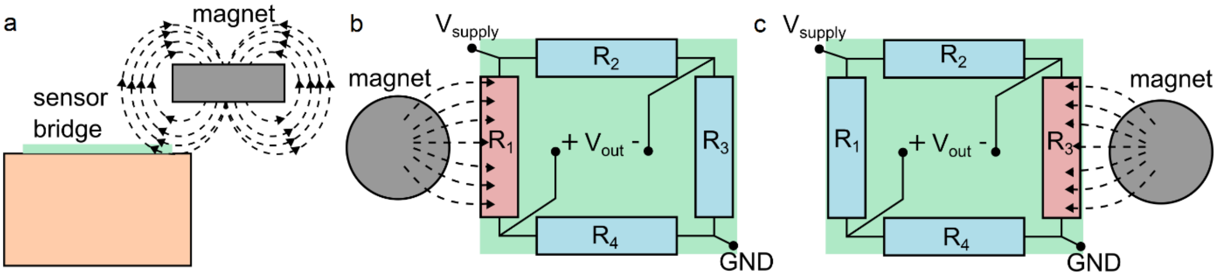

Figure S11. a) Schematics showing a side view of the location of the permanent magnet (gray rectangle with field lines) with respect to the Wheatstone bridge (green rectangle). The panels **b**) and **c**) demonstrate, which magnetoresistors are affected predominantly for two different positions of the permanent magnet.

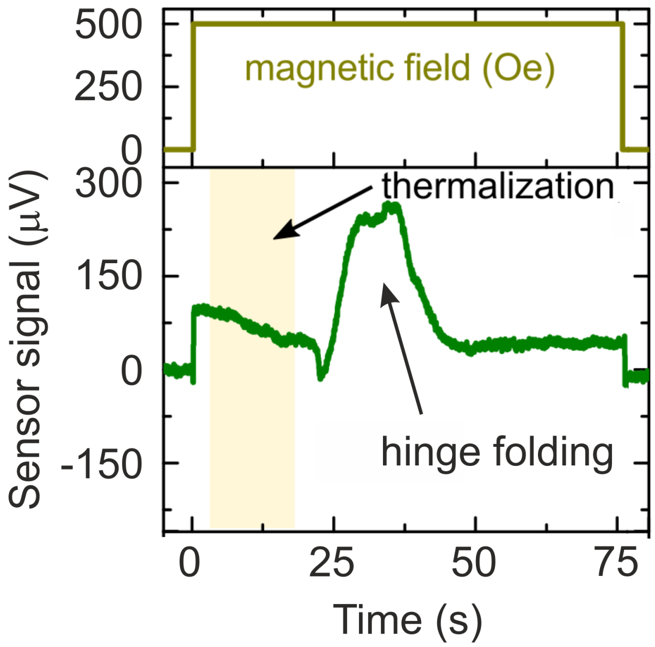

Figure S12. Time evolution of the anomalous Hall effect sensors when exposed to the magnetic field and illumination. The increased temperature from photothermal heating (Figure S7, Supporting Information) results in thermal drift of the sensor response.
